# Unraveling the structures, functions and mechanisms of epithelial membrane protein family in human cancers

**DOI:** 10.1186/s40164-022-00321-x

**Published:** 2022-10-10

**Authors:** Nan Zhang, Hong‑Ping Zhu, Wei Huang, Xiang Wen, Xin Xie, Xian Jiang, Cheng Peng, Bo Han, Gu He

**Affiliations:** 1 State Key Laboratory of Southwestern Chinese Medicine Resources, School of Pharmacy, Hospital of Chengdu University of Traditional Chinese Medicine, Chengdu University of Traditional Chinese Medicine, Chengdu, 611137 China; 2 Department of Dermatology, West China Hospital, Sichuan University, Chengdu, 610041 Sichuan China; 3 Laboratory of Dermatology, Clinical Institute of Inflammation and Immunology (CIII), Frontiers Science Center for Disease-Related Molecular Network and State Key Laboratory of Biotherapy, West China Hospital, Sichuan University and Collaborative Innovation Center of Biotherapy, Chengdu, 610041 China; 4 Antibiotics Research and Re‑Evaluation Key Laboratory of Sichuan Province, Sichuan Industrial Institute of Antibiotics, Chengdu University, Chengdu, 610106 China

**Keywords:** Epithelial membrane protein, Peripheral myelin protein 22, Cancer, Mutation, Programmed cell death

## Abstract

Peripheral myelin protein 22 (PMP22) and epithelial membrane proteins (EMP-1, -2, and -3) belong to a small hydrophobic membrane protein subfamily, with four transmembrane structures. PMP22 and EMPs are widely expressed in various tissues and play important roles in cell growth, differentiation, programmed cell death, and metastasis. PMP22 presents its highest expression in the peripheral nerve and participates in normal physiological and pathological processes of the peripheral nervous system. The progress of molecular genetics has shown that the genetic changes of the *PMP22* gene, including duplication, deletion, and point mutation, are behind various hereditary peripheral neuropathies. EMPs have different expression patterns in diverse tissues and are closely related to the risk of malignant tumor progression. In this review, we focus on the four members in this protein family which are related to disease pathogenesis and discuss gene mutations and post-translational modification of them. Further research into the interactions between structural alterations and function of PMP22 and EMPs will help understand their normal physiological function and role in diseases and might contribute to developing novel therapeutic tools.

**Supplementary Information:**

The online version contains supplementary material available at 10.1186/s40164-022-00321-x.

## Introduction

The growing number of cases and deaths of malignant tumors worldwide has increased the need for more effective diagnosis and treatment methods. Tumor pathogenesis is complex because tumors are heterogeneous and have high metastatic capabilities. Thus, it is necessary to comprehensively study the specific mechanisms of tumor development to provide effective diagnostic markers and new therapeutic targets. Currently, studies have identified several oncogenes and tumor suppressor genes (TSGs) that play key roles in tumor development, and effective anti-tumor therapy methods have been developed for these targets. However, gene variation and individual differences in tumors can impair current treatment methods and the wide range of therapeutic effects. Various proteins expressed on the cell membrane, including the growth arrest-specific 3(GAS3)/ peripheral myelin protein 22 kDa (PMP22) family, can participate in tumor cell proliferation, invasion, metastasis, and differentiation and have become novel biomarkers for tumor diagnosis and targeted therapy [1].

The (GAS3)/PMP22 family consists of at least seven members, namely PMP22, EMP1 (TMP), EMP2(XMP), EMP3(YMP), PERP, BCMP1, and lens fiber membrane intrinsic protein MP20. The amino acid sequences of these proteins have 30–40% homology, and their structures also share some similarities. GAS3/PMP22 family members are usually closely related to the development of different diseases, including inherited disorders of the peripheral nervous system (PNS) and other diseases related to the disorder of cell growth, differentiation, and apoptosis. Moreover, PMP22 mutations can induce neurodegenerative diseases, and the abnormal expression of EMPs might be related to cancer progression. Despite increasing research into the biological activities of PMP22 family members, the molecular mechanisms behind the pathological foundation of various diseases remains unknown [2]. Herein, we mainly summarized the structure, mutation, and modification sites of PMP22, EMP1, EMP2, and EMP3, as well as the diseases related to these proteins, especially cancer types.

## PMP22 protein family: genes, proteins, and structure

The exon–intron structure of the *PMP22* gene is highly conserved compared to EMPs [3]. The positions of introns are completely conserved between genes of the PMP22 protein family, corroborating that they belong to the same family and might have evolved from a common ancestor gene.

GAS3/PMP22 family members are often associated with the emergence of various diseases. For example, PMP22 mutations can induce neurodegenerative diseases, and abnormal expression of EMP3 can affect the occurrence of brain, breast, and T cell tumors. The amino acid sequences of the hydrophobic regions of PMP22 and EMPs proteins are highly conserved, especially in the two and four transmembrane domains, indicating that they are essential for the function of these proteins across the family [2, 4].

### Structure of genes and proteins

**Fig. 1:**
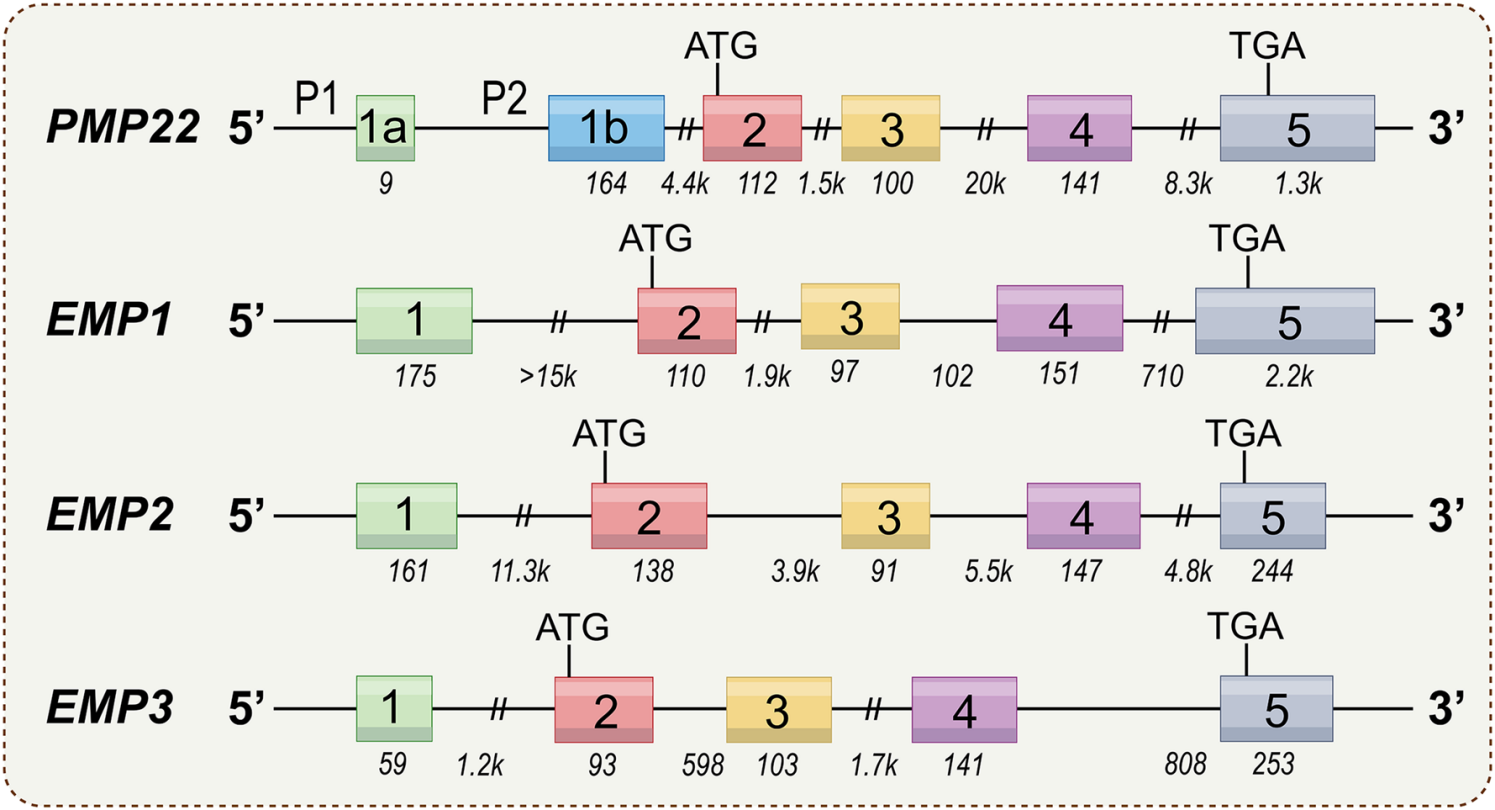
Schematic view of the genomic structure of PMP22 gene family. The boxes mark the exons. Start and stop codons are indicated. The numbers on the genes refer to exons. The italic numbers below the genes indicate the sizes of the introns and exons. The PMP22 gene generates two transcripts through alternative splicing of exons la and lb. These two transcripts are regulated by two alternative PMP22 promoters, P1 and P2

PMP22, the growth arrest-specific gene, was first isolated from growth-inhibited NIH3T3 cells. The *PMP22* gene is located in the 11.2 regions of the short arm of chromosome 17 (17p11.2), comprehending spans about 40 kb. It contains six exons at the 5′ end, four coding and two non-coding exons (Fig. 1). P1 and P2 promoters selectively transcribe exons 1a and 1b, leading to two distinct transcripts by alternative splicing to regulate PMP22 expression. The transcriptional protein of Exon 1a is mainly located in the peripheral nerve myelin sheath, while the transcriptional protein of Exon 1b is mainly in non-neural tissue. Exon 2 encodes the first transmembrane domains on the N terminus of PMP22, whereas Exon 3 encodes the first extracellular loop with a glycosylation site. Exon 4covers the second and half of the third transmembrane domains. The remaining third transmembrane domain, the second extracellular loop, the fourth transmembrane domain, and the 3' UTR are encoded and translated by Exon 5 [3, 5].

Furthermore, the *PMP22* gene encodes a transmembrane glycoprotein composed of 160 amino acids with a relative molecular weight of about 22 kDa. This protein is the main constituent of peripheral nerve myelin, and its amino acid sequence is conserved across species. As a highly conserved glycoprotein with four transmembrane domains, PMP22 is highly expressed in Schwann cells, accounting for 2–5% of all myelin proteins. PMP22 has an N-myristoylation region, an N-glycosylation region, a casein kinase II phosphorylation site, and a protein kinase C phosphorylation site [2, 4].

Moreover, the *EMP1* gene is located on the long arm of chromosome 12 (12p12.3) and encodes a glycoprotein containing four transmembrane regions [6, 7]. The *EMP1* gene generates a 2.7 kb primary transcript in all tissues that express it, with five exons: 0.22, 0.12, 0.1, 0.14, and 2.2 kb. The interval length of each exon region is 1.5, 1.9, 0.1, and 0.7 kb (Fig. 1) [3].

EMP1 is a hydrophobic four transmembrane transporter with 160 amino acid residues and 40% sequence homology with PMP22. It primarily regulates epithelial cell growth and differentiation. The EMP1 protein has an N-myristoylation region, a casein kinase 2 phosphorylation area, and an N-glycosylation region, but unlike PMP22, no reports have detected a protein kinase C phosphorylation site. EMP1 can regulate cell proliferation and differentiation, especially in epithelial cells and various malignant tumors [2, 8].

EMP1 is highly expressed in gastric cancer, glioma, early neurons, and immature neurons. In contrast, EMP-1 transcript levels are the lowest in oral cancer, laryngeal, gastric, and esophageal cancers [1].

The human *EMP2* gene is located on chromosome 16 (16p13.13) [6], and its expression is tissue-specific, with the highest expression in the lung and only very low in the breast. EMP2 can mainly affect the progression of B-cell tumors and stress-induced apoptosis.

Moreover, EMP2 and PMP22, like EMP1, share roughly 40% of the amino acid sequence. The EMP2 protein has three N-linked glycosylation sites on its first extracellular loop, a casein kinase two phosphorylation region, and a protein kinase C phosphorylation site, with no N-myristoylation region [2].

Transmembrane proteins often aid in the formation of protein signaling complexes. Previous studies have shown that EMP2 regulates related cell signaling pathways by interacting with integrins, caveolin, and other cell signaling pathways, to regulate cellular processes [8–10].

According to the Human Protein Atlas, EMP2 mRNA, similar to other tetraspan proteins, has a diverse expression profile, highly expressed in the lung and moderately expressed in the eyes, thyroid, heart, intestine, and uterus. EMP2 expression mutations are also associated with diseases, including childhood nephrotic syndrome for patients carrying 21 C > G (p.phe7leu) mutations. Nonetheless, EMP2 protein levels are more discrete than mRNA levels, which limits the expression in type 1 lung cells, endometrium, keratinocytes in the skin, and numerous tissues, including retinal pigment epithelium, ciliary body, and corneal epithelium of the eye. The *EMP3* gene is mapped to chromosome 19 (19q13.3) and has a 30% homologous region with PMP22, EMP1, and EMP2 [6].

EMP3 consists of 165 amino acids, four transmembrane domains, and two extracellular loops. The first extracellular loop has N-glycosylation sites. There are also protein kinase C phosphorylation sites and an N-myristoylation region on EMP3, but no casein kinase 2 phosphorylation site [2]. Additionally, EMP3 is highly expressed in peripheral blood granulocytes, ovary, rectum, embryonic lung, liver, and kidney [8].

### Missense substitution of the PMP22 gene family

High-throughput sequencing technology can detect many genomic variations linked to disease susceptibility or drug reactivity. The most common genetic differences in the human genome are single nucleotide variations (SNVs) [11], which are most concerning in protein-coding regions. The SNV in the coding region is considered synonymous when there is no amino acid change. If the replacement leads to a protein sequence change, the SNV is considered non-synonymous. Non-synonymous mutations can be further divided into: missense mutations that lead to changes in single amino acid, or nonsense mutations that produce truncated or longer proteins. Missense mutations, protein variants with single amino acid variation (SAVs), are essential in biomedicine, because even a single amino acid substitution might induce drastic structural changes, compromising protein stability, or key structural alterations that might interfere with the binding interface, finally affecting protein function [12]. Herein, we collected information about gene missense mutation of PMP22 family members from database. The most representative mutations are summarized in Additional file 1: Table S1.

**Fig. 2:**
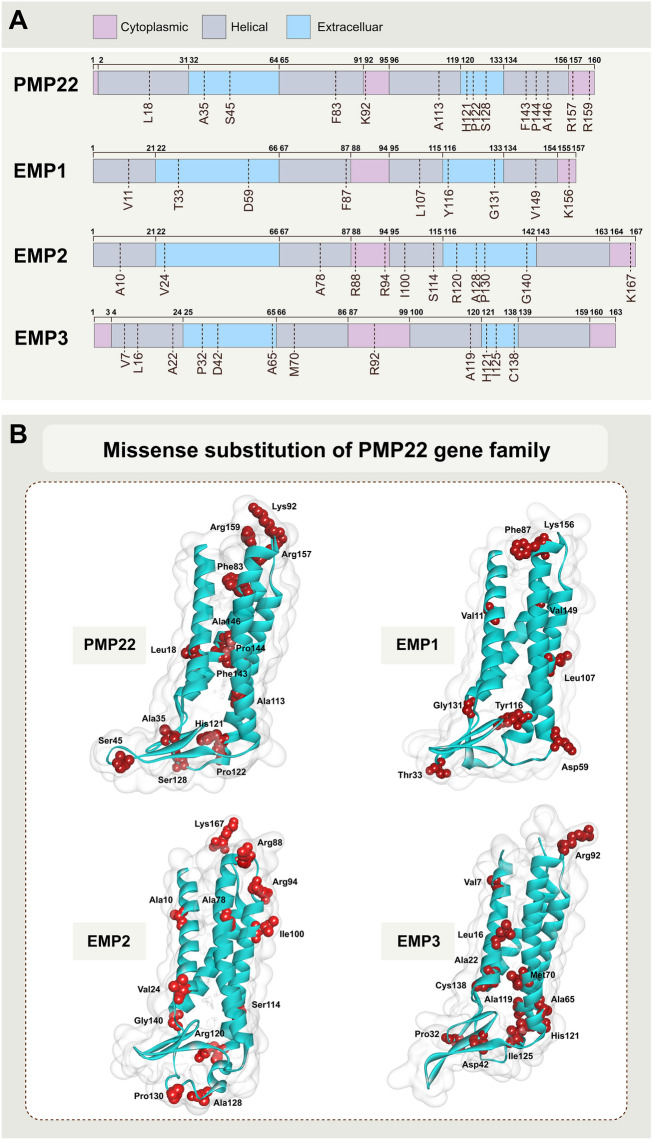
Missense substitution of PMP22 protein family. **A** Schematic view of structure of the PMP22 family of proteins. The pink area represents the cytoplasmic domain, the gray represents the transmembrane domain, and the blue represents the extracellular domain. The upper numbers refer to the starting amino acid sites of different domains, and the lower dotted line refers to the mutated amino acid sites. **B** Missense substitution of PMP22 protein family. The protein structures were modelled by AlphaFold2 [13]. Only the sites with mutation frequency greater than 2 were shown in the data

There are 14 sites with missense mutation frequency ≥ 2 in the *PMP22* gene, three sites in the cytoplasmic region of the PMP22 protein, six in the transmembrane region, and five in the extracellular region. There are fewer missense mutations with frequency ≥ 2 in the *EMP1* gene. They mainly include nine sites, one located in the cytoplasmic region of EMP1, four in the transmembrane region, and four in the extracellular region of the EMP1 protein. There are 12 sites with missense mutation frequency ≥ 2 in the *EMP2* gene, three located in the cytoplasmic region of the protein, four in the transmembrane region, and five in the extracellular region of the protein. There are 12 additional sites where the missense mutation frequency ≥ 2 of *EMP3*, one in the cytoplasmic area, five in the transmembrane region, and six in the extracellular portion of the protein (Fig. 2).

### Post-translational modifications of the PMP22 protein family

**Fig. 3:**
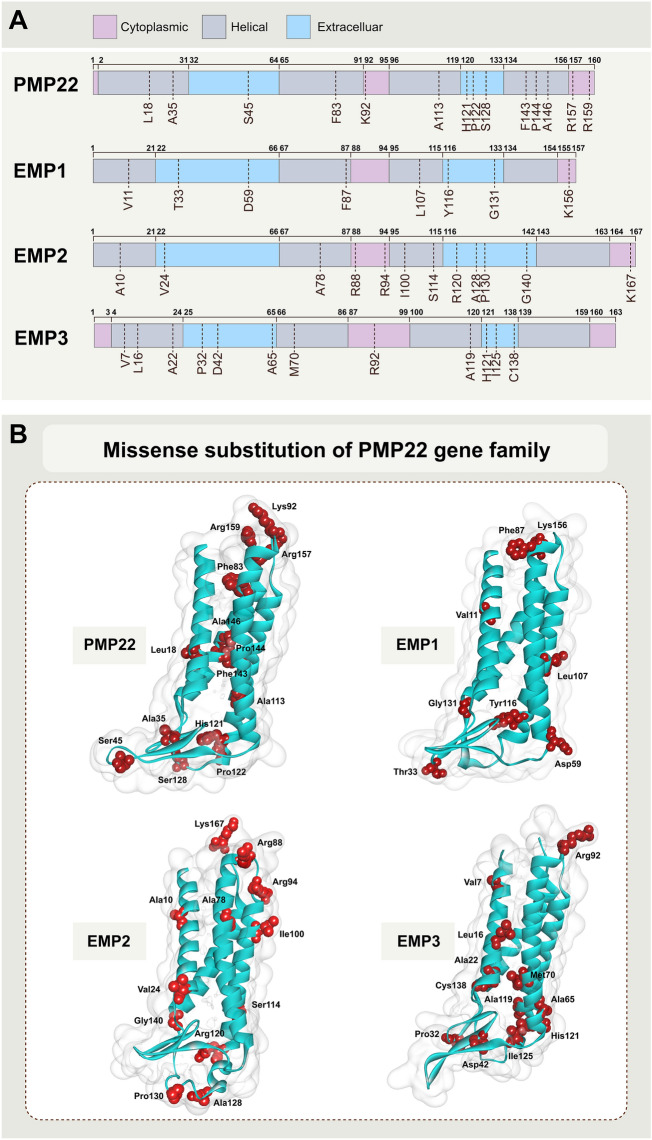
Post-translational modifications of PMP22 protein family. **A** Schematic view of structure of the PMP22 family of proteins with post-translational modification sites. The upper numbers refer to the starting amino acid sites of different domains, and the lower dotted line refers to the mutated amino acid sites. **B** Post-translational modification sites of PMP22 protein family. The protein structures were modelled by AlphaFold2 [13]

Post-translational modifications (PTMs) are covalent modifications of proteins after synthesis, generally enzymatic modifications and can be produced by modifying small molecular substances. After synthesis, peptides are modified into mature proteins and play a crucial part in cellular biological processes. Therefore, as an essential part of the cell signaling pathways, PTMs are the molecular basis of protein dynamic response and interaction, and an important target of cell signal regulation. Proteins can have various PTMs, changing their structure and function to variable degrees, resulting in a sequence of effects on physiological activities (Fig. 3) [14].

#### N-Glycosylation and phosphorylation

PMP22 family members have high domain homology, and each member has at least one N-glycosylation and phosphorylation region. Glycosylation comprehends the addition of sugars to proteins or lipids by glycosyltransferases, and occurs in the endoplasmic reticulum and Golgi apparatus. Glycosylations are an important post-translational modification, and most membrane and secretory proteins are glycoproteins. Glycosylations not only affect the spatial conformation, activity, transportation, and localization of proteins [15] but also play a critical role in signal transduction, molecular recognition, and immunity [16]. N-acetylglucosamine (GlcNAc) and mannose are connected to Asn residues by glycosidase to produce the basic framework of N-glycan. Galactose (Gal), N-acetylneuraminic acid (Neu5Ac), galactosamine (GalN), and other molecules can be involved in the whole sugar chain formation process. Glycosylation is often relevant for the function of membrane proteins [17–19].

**Table 1:** Protein post translation modifications of epithelial membrane protein family members

UniProt No.	Protein name	Position	Sequence window	Modification
P54849	EMP1	43	ASVGLWK**N**CTNISCS	N-Glycosylation
P54849	EMP1	46	GLWKNCT**N**ISCSDSL	N-Glycosylation
P54849	EMP1	62	YASEDAL**K**TVQAFMI	Methylation
P54849	EMP1	33	NVWLVSN**T**VDASVGL	Phosphorylation
P54849	EMP1	52	TNISCSD**S**LSYANED	Phosphorylation
P54849	EMP1	55	SCSDSLS**Y**ANEDALK	Phosphorylation
P54849	EMP1	57	SDSLSYA**S**EDALKTV	Phosphorylation
P54849	EMP1	126	HYANRDG**T**QYHHSYS	Phosphorylation
P54849	EMP1	131	DGTQYHH**S**YSYILGW	Phosphorylation
P54849	EMP1	133	TQYHHGY**S**YILGWIC	Phosphorylation
*P54851*	*EMP2*	*44*	*DVWRICT****N****NTNCTVI*	*N-Glycosylation*
*P54851*	*EMP2*	*47*	*RICTNNT****N****CTVINDS*	*Glycosylation*
*P54851*	*EMP2*	*52*	*CTVI****N****DSFQEYS*	*N-Glycosylation*
*P54851*	*EMP2*	*116*	*VMIAASI****Y****TDRREDI*	*Phosphorylation*
*P54851*	*EMP2*	*129*	*DIHDKNA****K****FYPVTRE*	*Ubiquitylation*
*P54851*	*EMP2*	*129*	*DIHDKNA****K****FYPVTRE*	*Acetylation*
*P54851*	*EMP2*	*141*	*TREGSYG****Y****SYILAWV*	*Phosphorylation*
*P54851*	*EMP2*	*142*	*REGSYGY****S****YILAWVA*	*Phosphorylation*
P54852	EMP3	1	**M**SLLLLVVS	Acetylation
P54852	EMP3	2	M**S**LLLLVVS	Phosphorylation
P54852	EMP3	9	SLLLLVV**S**ALHILIL	Phosphorylation
P54852	EMP3	23	LILLFVA**T**LDKSWWT	Phosphorylation
P54852	EMP3	46	WYDCTW**N**NDTKTWACS	N-Glycosylation
P54852	EMP3	47	WYDCTWN**N**DTKTWAC	N-Glycosylation
P54852	EMP3	56	TKTWACS**N**VSENGWLK	N-Glycosylation
P54852	EMP3	128	HAEEILE**K**HPRGGSF	Methylation
*Q01453*	*PMP22*	*41*	*ATDLWQ****N****CSTSSSGNV*	*Glycosylation*
*Q01453*	*PMP22*	*99*	*KGGRFYITGIFQILA*	*Phosphorylation*
*Q01453*	*PMP22*	*117*	*VMSAAAI****Y****TVRHPEW*	*Phosphorylation*
*Q01453*	*PMP22*	*118*	*MSAAAIY****T****VRHPEWH*	*Phosphorylation*
*Q01453*	*PMP22*	*153*	*ALLSGVI****Y****VILRKRE*	*Phosphorylation*

Bold letters represent modified amino acid residues. Italic entries are only used to help distinguish which protein is corresponding to the information in this area.

The phosphorylation region includes the protein kinase C phosphorylation and casein kinase 2 (CK2) phosphorylation regions [14, 20–22]. PKC can phosphorylate hydroxyl groups of serine and threonine residues of interacting proteins. The cellular processes regulated by PKC include cell proliferation, survival, invasion, metastasis, apoptosis, and tumorigenesis. Changes in PKC expression and activity changes are common in human cancers [23]. Additionally, PKC can cooperate with various oncogenes, such as Ras, Fos, and myc, and serve as an important member of the cancer signaling pathway [24]. Therefore, PKC has been investigated as a possible target for cancer treatment. CK2 is a highly conserved serine/threonine kinase and participates in diverse cellular functions, including cell viability maintenance [25, 26]. Moreover, most human cancers seemingly perform high CK2 activity, suggesting that it might be involved in cell proliferation and prevent apoptosis. Therefore, understanding the role of phosphorylation modification sites in the biological properties of PMP22 family members is crucial. The N-glycosylation and phosphorylation modification sites of PMP22 protein family members were summarized by investigating the databases related to PTMs. PMP22 contains one N-glycosylation site at position 41(asparagine) and four phosphorylation sites (T99, Y117, T118, and Y153). EMP1 has two N-glycosylation sites (N43 and N46) and seven phosphorylation sites (T33, S52, Y55, S57, T126, S131, and S133). EMP2 has three N-glycosylation sites (N44, N47, and N52) and three phosphorylation sites (Y116, Y141, and S142). EMP3 has three N-glycosylation sites (N46, N47, and N56) and three phosphorylation sites (S2, S9, and T23) (Table 1).

#### Methylation, acetylation, and ubiquitylation

Protein methylation is a universal PTM with an indispensable regulatory role in cell physiology and pathogenesis [27]. Protein methylation refers to the transfer of methyl to a protein residue. S-adenosylmethionine (SAM) is the protein methylation donor, and the receptors are usually lysine-guanidine amino groups and arginine [28]. Furthermore, methylation might occur in histidine's imidazole group, glutamine's and asparagine's amide sites, cysteine's sulfhydryl group, cysteine's carboxyl group, and glutamic acid's and aspartic acid's side-chain carboxyl groups [27, 29, 30]. Besides histones, many proteins can be methylated. Protein methylation can impact protein–protein interactions, protein-DNA or protein-RNA interactions, protein stability, subcellular localization, and enzyme activity [28]. Additionally, methylation of many transcription factors, co-regulators, RNA poly merase II and histones can recruite some effector proteins contain tudor domain to the transcriptional start sites (TSSs), resulting in affecting gene transcription and expression [29]. Methylation can also influence the association of some arginine-containing proteins with nucleic acids. Some arginine-containing proteins rich in repeating RGG and RG motifs, which are found to be the RNA binding segments in proteins. The different degrees methylation of RGG/RG regions may regulate the interactions with RNA [28]. Methylation can appear at different amino acid sites, while the same amino acid might exist at more than one methylation modification site. Therefore, determining methylation sites is the primary task in studying protein functions. Understanding the effects of protein methylation and further researching its interactions with other PTMs, combined with high throughput proteomics methods and other molecular biology experimental approaches, can help better understand the role of PTMs in cell signaling pathways, animal development, and disease diagnosis and therapy. Furthermore, intervention in key links will help provide a strong scientific basis and measures for preventing and treating related diseases. We found that PMP22 protein family members have few methylations, with only one methylation site in EMP1 and EMP3, located in lysine 62 and 128, respectively (Table 1).

Acetylation modification is a reversible and evolutionarily conserved PTM that functions in transcriptional regulation and signal transduction in eukaryotic and prokaryotic cells. Early studies have found that acetylations mainly occur on histones in the nucleus and are involved in regulating gene transcription [31]. Later, with the improved recognition sensitivity of protein mass spectrometry, more non-histone proteins were found to be regulated by acetylations, comprehending the main part of acetylated proteins in mammalian cells [31]. Non-histone acetylation involves key physiological and disease-related cellular processes, such as gene transcription, signaling pathway regulation, metabolic regulation, and protein stability regulation [27]. Acetylation modification regulates plenty of metabolic enzymes, including all of the glycolysis enzymes, and many critical metabolism related transcription factors, thereby plays a complex role in regulating cancer metabolism [27]. Acetylation alters protein function via diverse mechanisms, including protein stability, enzyme activity, subcellular localization, cross-talk with other PTMs, and protein–protein and protein-DNA interactions. The dynamic transition between phosphorylation and dephosphorylation of tumor suppressor gene Rb affects the cell cycle. Acetylation of Rb inhibits its phosphorylation, affects its nuclear localization, resulting in an impact on cell proliferation and tumor progression. Proteins with bromodomians contains acetyl-bingding modules performed important roles in DNA damage response. Some of the proteins alter subcellular localization when DNA damage happens by regulate their acetylation level. In the PMP22 family, acetylation is relatively simple. Only lysine 129 in EMP2 and methionine 1 in EMP3 can be acetylated (Table 1).

Similar to other PTMs, ubiquitination is also a strictly regulated reversible process [32–34]. The ubiquitin-proteosome system is responsible for most protein degradation in eukaryotic cells. Moreover, ubiquitination can directly affect the activity and localization of proteins and regulated various cell physiological activities to maintain cell homeostasis, such as cell cycle, cell survival and apoptosis, transcriptional regulation, DNA damage repair, and immune response [35]. Numerous PTMs in the same protein increase complexity in the life process. Generally, acetylation-dependent protein stability mechanism prevents the protein from ubiquitination and proteasome degradation. Acetylation and ubiquitination can compete for the same lysine site. For example, P300 can mediate the acetylation of Smad7 Lys64 and Lys70 sites and prevent Smad7 from ubiquitination by ubiquitin regulator 1 (Smurf1) and avoid degradation [36]. On the other hand, acetylation can accelerate protein degradation by increasing ubiquitination. For example, acetylation of Pepck1 can recruit the E3 ubiquitin ligase ubr5, resulting in Pepck1 degradation [37]. DNMT1 acetylation can promote the ubiquitination of DNMT1 by UHRF1, eventually leading to DNMT1 degradation [38]. The crosstalk between different PTMs can regulate the function of proteins in certain cells or under different physiological and pathological conditions. By searching the PTMs database, we found that the lysine 129 in the EMP2 amino acid sequence can be acetylated or ubiquitinated (Table 1). However, there is still a lack of definite reports on whether this PTMs crosstalk on EMP2 protein is related to its complex function.

## Biological functions of the PMP22 protein family

The GAS-3/PMP22 gene family of tumor-associated membrane proteins (TMPs) has multiple biological functions and plays an important role in tumor development. The biological functions of the PMP22 protein family are described below.

### PMP22

The PMP22 protein regulates cell growth cycle, cell adhesion, and apoptosis, and is irregularly expressed in various malignant tumors. Its overexpression is associated with the occurrence and progression of lung cancer, breast cancer, gastric cancer, pancreatic cancer, and osteosarcoma [39, 40]. Hence, PMP22 might have a great value in the clinical treatment and prognosis of malignant tumors.

Multiple studies have shown that PMP22 has complex regulations, including transcriptional by intricate transcriptional regulatory elements and post-transcriptional regulations [5, 41]. Although more than 90% of the protein synthesized is rapidly degraded before reaching the Schwann cell plasma membrane, PMP22 is widely expressed in Schwann cells, and a few newly synthesized PMP22 are permitted transport to the cell surface benefit from the complex glycosylation [5]. PMP22 is highly expressed in the compact portion of myelin in peripheral nervous and the genetic alterations of *PMP22* gene is harmful to the normal physiological function of peripheral nervous system. Mutations or deletions in different parts of the *PMP22* gene can lead to hereditary peripheral nervous system diseases, including Charcot Marie Tooth disease (CMT), and hereditary neuropathy with liability to pressure palsies (HNPP) [4, 42, 43]. The transcripts of PMP22 are most abundantly expressed in myelinated Schwann cells of the peripheral nervous system, but it is also expressed in some non-neural cells and tissues, such as fibroblasts, vascular endothelial cells, and intestinal, pulmonary, and choroid plexus epithelial cells [44]. As a member of the growth inhibition-specific gene family, PMP22 plays a vital role in cell proliferation and morphology control. PMP22 has been more examined in genetic diseases of the peripheral nervous system but less in tumors. Thus, its expression, function, and mechanism in malignant tumors remain poorly understood.

#### Regulation of cell growth cycle, adhesion, proliferation, apoptosis and invasion by PMP22

Numerous studies have shown that PMP22 is critical in regulating cell growth, adhesion, and apoptosis. In Schwann cells, high PMP22 expression decrease the incorporation of 5-Bromo-2'-deoxy-uridine (BrdU) which may lead to a reduction of related RNA formation, prevents cells from entering the S + G2/M phase from the G0/G1 phase. In contrast, inhibiting PMP22 expression can increase DNA synthesis, causing cells to enter the S + G2/M phase. Thus, PMP22 negatively regulates the cycle of Schwann cells [45]. The extracellular region of PMP22 contains an N-terminal linked glycosylation sequence, which carries the L2/HNK-1 epitope after carbohydrate modification [46]. HNK-1 can confirm carbohydrate-structured cell surface glycoproteins such as myelin P0 protein, neural adhesion protein N-CAM, myelin protein Mac, certain glycolipids, and proteoglycans. The above proteins are related to cell adhesion, cell–cell interaction, and cell and external matrix interaction. The correlation between PMP22 and these proteins suggests that PMP22 might also be related to cell adhesion. According to research on PMP22 in human endometrial, overexpression of PMP22 significantly increased the expression of integrin α6 on the cell surface and promoted the binding of PMP22 to extracellular matrix adhesion protein, while the inhibition of PMP22 expression decreased integrin α6 and interfere with the binding of PMP22 to extracellular matrix layer adhesion protein. And the expression activity of PMP22 in proliferative endometrium was much greater than in secretory endometrium, indicating that PMP22 may be associated with regulating cell adhesion and cell proliferation [47].

Upregulation of PMP22 in NIH3T3 fibroblasts can also generate a substantial number of apoptosis, which Bcl-2 and protease inhibitors could block. These results showed that PMP22 overexpression could cause fibroblast death via apoptosis [48]. In NIH3T3 fibroblasts, the expression of PMP22 significantly increases when serum starvation-induced cell growth is inhibited. Further studies have shown that it causes slow cell growth, decreases migration ability, and even apoptosis, mainly through the Rho GTPase signaling pathway, which critically influences on cell deformation and migration [49].

Other studies have found that PMP22 is an important constituent of the intercellular junction in epithelial cells and endothelial cells and is co-located with tight junction protein 1 (ZO-1) and closure protein occludin at the cell tight junction or adjacent. Additionally, PMP22 overexpression promotes the formation of intercellular junctions in ZO-1 positive cells in fibroblasts. Low expression or deletion of PMP22 can obstruct tight junction formation between cells, reduce cell adhesion, and increase cell migration [50]. PMP22 is a binding partner in the integrin / laminin complex, and forms complex with integrin α6β4 and affects the connection between myelin Schwann cells (SCs) and basal layer [51].In the scratch healing experiment with epithelial cells, researchers found that the migration ability of PMP22 overexpressing cells was prominently lower than controls. PMP22 is significantly expressed in tumor microenvironment macrophages, secretes cell migration factors, and promotes tumor migration.

**Fig. 4:**
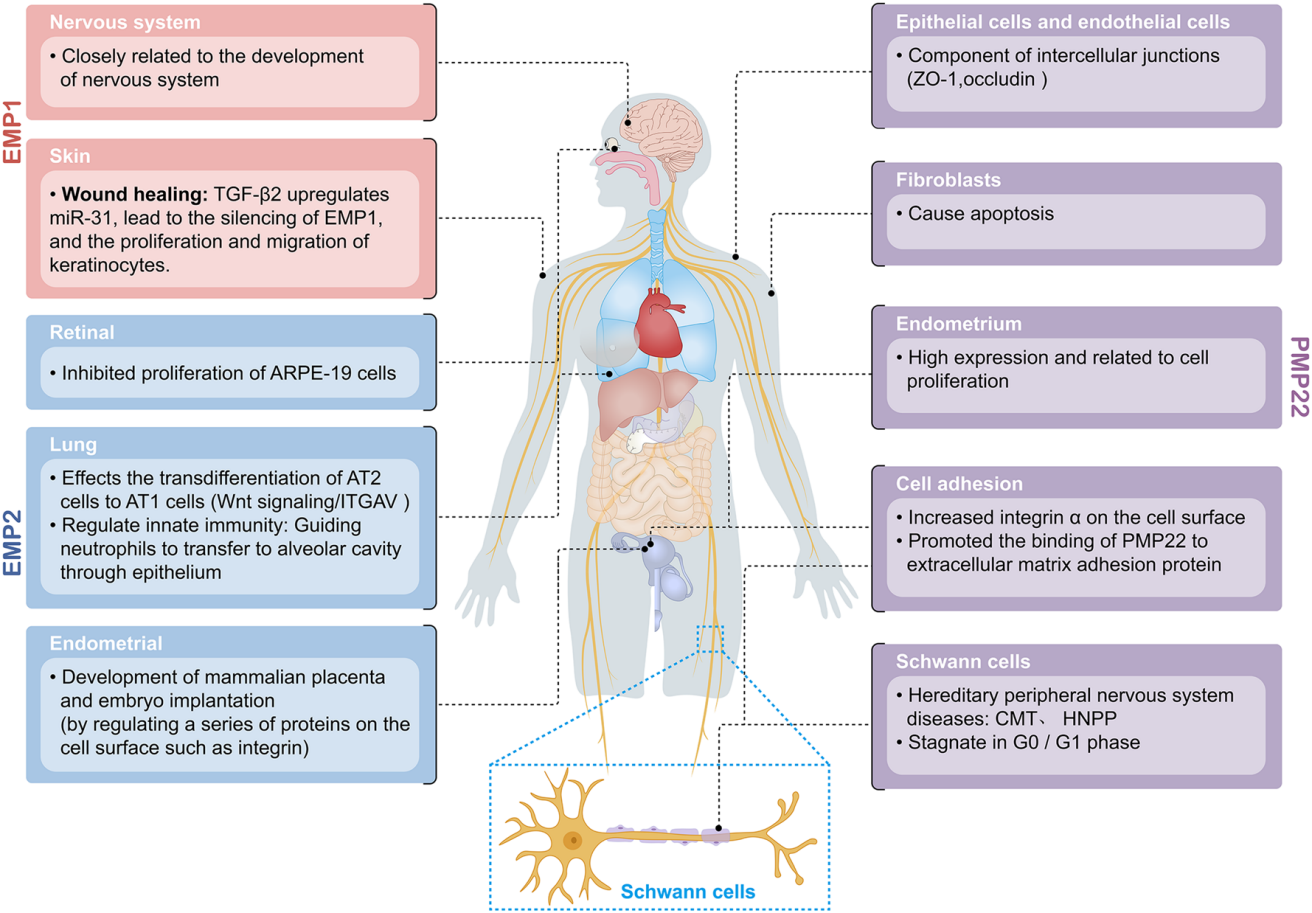
Function of PMP22 protein family in normal tissues

Before apoptosis or cell morphological changes, ADP ribosylation factor 6 (Arf-6)-positive plasma membrane endosomes phagocytose exogenous PMP22 vacuoles, important to regulate actin skeleton, cell polarity, cell adhesion, and cell migration. Overall, PMP22 has complex functions regulating intercellular connectivity, invasion, and morphological changes (Fig. 4) [52].

#### PMP22 in cancer

**Fig. 5:**
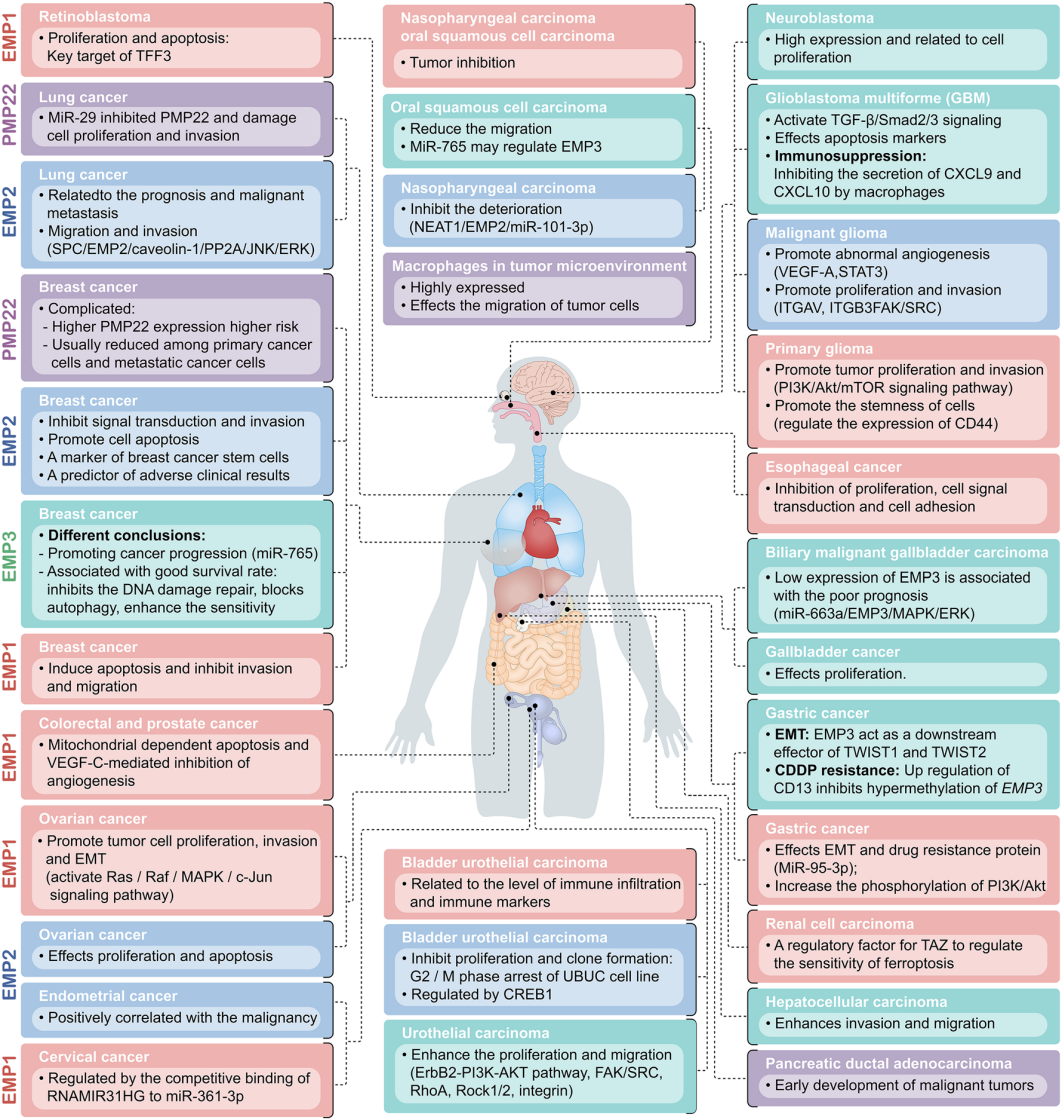
Function of PMP22 protein family in tumor tissues

Although *PMP22* gene deletion has different specific effects on tumor proliferation and demyelination, it also has specific effects on cancer and peripheral tissue cells. In the proliferation experiment with highly metastatic osteosarcoma cell lines, the proliferation ability of osteosarcoma cells successfully transfected with PMP22 was significantly higher than controls [40, 53]. PMP22 is overexpressed in gastric cancer cells and tissues. Knockdown of PMP22 inhibited cell growth. PMP22 enhanced the inhibition of p53 transcriptional activity and down regulated p53 targeted genes. In gastric cancer cells, there is a conserved miRNA recognition site of miR-139-5p on the 3 'UTR of PMP22. MiR-139-5p targets NF-κB signal pathway negatively regulates PMP22 and gastric cancer cell proliferation. *PMP22* gene expression was shown to be reduced in an animal model of lung cancer, whereas other investigations on lung cancer cells revealed PMP22 transcript amplification. The inhibition of PMP22 expression by miR-29 in lung cancer cells damages cell proliferation and metastasis, indicating that PMP22 is related to cancer progression [54]. PMP22 mRNA is also higher in pancreatic ductal adenocarcinoma than in normal pancreatic tissue, suggesting that it is involved in the early development of malignant tumors [55]. The analysis of *PMP22* gene expression in breast cancer is also very complicated [56]. An earlier study compared the invasiveness and noninvasiveness of human breast cancer cell lines with normal breast epithelial cells and found that the PMP22 mRNA level increased. To further evaluate the effects of PMP22 differential expression on the prognosis of breast cancer, the authors concluded that patients with higher PMP22 expression had a higher risk of dying from cancer than those with the same clinical covariates but with lower PMP22 expression. However, a recent investigation of the differential gene expression profiles of normal, primary cancer, and metastatic cancer cells showed that PMP22 expression is often reduced [57–59]. The differential expression of PMP22 mRNA in different tumors indicates that to better understand its role in the tumor, PMP22 protein should be more thoroughly studied, which might involve the post-transcriptional regulation of the *PMP22* gene. Furthermore, there is no substantial difference in the PMP22 mRNA level in colon cancer compared to normal tissues. However, PMP22 protein levels are significantly lower in colon cancer samples than in normal colonic tissues, possibly due to the low post-translational efficiency of PMP22 mRNA in these tissues and cells (Fig. 5).

### EMP1

EMP1 is a membrane glycoprotein. The EMP1 mRNA levels are the highest in the esophagus, followed by the gallbladder and adipose tissue. Meanwhile, the EMP1 protein levels are the highest in the stomach. EMP1 is highly correlated to the nervous system's development and regulates the cell cycle, death, and interaction. PMP22 inhibits EMP1 expression in fibroblasts and Schwann cells [1]. EMP1 might potentially be involved in skin wound healing. Highly expressed transforming growth factor β2 (TGF-β2) in skin wounds can upregulate miR-31 in keratinocytes, silencing the miR-31 target protein EMP1 and enhancing the proliferation and migration of keratinocytes [60].

Similar to PMP22, EMP1 has different mRNA and protein levels in diverse cancers and is involved in tumor cell proliferation, invasion, metastasis, and epithelial-mesenchymal transformation (EMT). The mRNA expression of EMP1 is upregulated in glioma and breast cancer, and associated with the expression of myc and ERBB2 receptors [61]. Increased EMP1 expression is also associated with increased tumor grade (*p* < 0.001) and poor prognosis (*p* < 0.001) in the TCGA, Rembrandt, and CGGA human glioma databases. In primary glioma cells, *EMP1* gene silencing can significantly inhibit tumor proliferation and invasion. Activating the PI3K/Akt/mTOR signaling pathway greatly triggers EMP1-promoted glioma progression. Additionally, EMP1 silencing inhibits the invasion and proliferation of glioma cells by inhibiting the PI3K-Akt signaling pathway [62]. CD44 is verified as a cell-surface biomarker of cancer stem cells (CSCs) and served as a crucial regulator for tumor development. The inappropriate regulation of CD44 would participate in formation of numerous cancers. Previous reports have shown that EMP1 can regulate CD44 expression in glioma stem cells, resulting in promoting of cancer cell stemness [63]. Pediatric leukemia patients with high EMP1 expression have a considerably poorer five-year survival rate [64]. The level of immune infiltration and a range of immune markers are also closely related to EMP1 expression in bladder urothelial carcinoma (BLCA) [65]. In ovarian cancer, EMP1 overexpression can activate the Ras/Raf/MAPK/c-Jun signaling pathway and promote tumor cell proliferation, invasion, and EMT [66].

Furthermore, EMP1 might be associated with tumor inhibition in other cancers such as nasopharyngeal carcinoma, oral squamous cell carcinoma, and prostate cancer. The EMP1 protein levels in nasopharyngeal carcinoma, prostate cancer, breast cancer, and colorectal cancer are significantly lower compared to normal tissues (*p* < 0.05). The decrease of EMP1 expression is significantly correlated with the T stage, lymph node metastasis, clinical stage, and histological grading of nasopharyngeal carcinoma (*p* < 0.05). The decreased expression of EMP1 in nasopharyngeal carcinoma, prostate cancer, breast cancer, and colorectal cancer cells can lead to a lower survival rate, a higher proportion of apoptosis, decreased migration and invasion abilities, and lower expression of vascular endothelial growth factor C protein (*p* < 0.05), indicating that EMP1 might play an important role as a negative regulator of these tumor cells [67–70]. Meanwhile, EMP1 has less research in breast cancer. The EMP1 protein level in breast cancer tissue samples significantly decreased and were significantly correlated with T stage, lymph node metastasis, and histopathologic grade. Enhancing EMP1 levels in breast cancer cells can induce apoptosis and inhibit invasion and migration [69]. In addition, multiple studies have shown that the levels of EMP1 protein are downregulated in esophageal cancer cells, the growth of esophageal cancer cells stably expressing EMP1 is slow, and genes related to cell signal transduction and cell adhesion are down-regulated [71]. Cell lines overexpressing EMP1 presented mitochondrial-dependent apoptosis and VEGF-C-mediated tumor angiogenesis inhibition in a colorectal and prostate cancer study [68, 70]. The expression of the *EMP1* gene is also significantly downregulated in cervical cancer biopsy tissues. The competitive binding of long noncoding RNAMIR31HG to miR-361-3p can regulate EMP1 expression, affecting the progression of cervical cancer [72]. In retinoblastoma, EMP1 might be the key target of tumor suppressor trefoil factor family peptide 3 (TFF3) signal transduction. The overexpression of TFF3 can induce up-regulation of p53 activity and miR-34a, reduce the expression levels of the miR-34a target protein EMP1, significantly inhibit the proliferation activity and increase the apoptosis of Y79 retinoblastoma cell [73]. A study on the development of drug resistance in gastric cancer using microRNAs (miRNAs) and cisplatin (DDP) showed that EMP1 was the target of miR-95-3p. The EMT and drug resistance protein were inhibited by miR-95-3p downregulation and increased PI3K/Akt pathway phosphorylation in gastric cancer [74].

Moreover, EMP1 is only rarely found in renal cell cancer. A study on the effect of Hippo pathway effector Yap (yes associated protein 1)/TAZ (transcriptional coactivator with PDZ binding motif) on the regulation of cancer cells on ferroptosis, a new programmed cell death, showed that EMP1 is a regulatory factor for TAZ to regulate the sensitivity of ferroptosis in renal cell carcinoma cells [75, 76].

### EMP2

#### Biological function of EMP2 in normal tissues

EMP2 is a tissue-specific protein expressed in the cell membrane and presents diversified expression patterns in different tissues and organs. Its expression levels are the highest in the lung, and only a very low level is detected in the breast. EMP2 might be crucial in a range of cells' physiological processes. Its overexpression in ARPE-19 (retinal pigment epithelium) cells causes collagen gel shrinkage in vitreous body retinopathy (PVR) [77]. High EMP2 levels also inhibit ARPE-19 cell proliferation. These results demonstrated the potential of EMP2 in controlling the biology of the retinal pigment epithelium. Additionally, EMP2 can critically regulate a series of effective molecular interactions required for embryo implantation, such as integrin, a heterodimer transmembrane protein closely related to cell proliferation, and intercellular and intracellular signal transduction [78, 79]. EMP2 is abundant in secretory endometrial and chorionic cells. During the window period of embryo implantation, EMP2 is expressed in cells and transferred to the top membrane surface of endometrial cells. It can regulate the development of the mammalian placenta and affect embryo implantation by regulating the expression of integrin such αv and β3 and many proteins on the cell surface [80].

Benefiting from the rapid development of gene chips and the support of diverse bioinformatics databases, the Gene Expression Omnibus (GEO) gradually provides excellent assistance for studying gene function. Several studies based on the GEO database, next-generation transcriptome sequencing, and comprehensive bioinformatics analysis demonstrated that EMP2 is differentially expressed in lung cancer and normal lung tissues, and its transcription levels are significantly correlated with the survival rate of the adenocarcinoma subgroup [81, 82]. However, the exact function of EMP2 in lung biology and its role in the carcinogenesis of lung cells remain un-clear.

The alveolar area accounts for more than 99% of the alveoli's large inner surface area. The enormous alveolar surface is susceptible to significant physiological renewal. Most the alveolar surface area is covered by flat type 1 alveolar epithelial cells (AT1), with the rest arranged by type II cells.AT1 cells, known for their solute transport and gas exchange, have little immune function. Type II cells synthesize, secrete, and circulate pulmonary surfactant, transport ions, participate in lung immune response, and act as progenitor cells in lung injury. Moreover, AT1 cells die through apoptosis after injury, leaving an exfoliated alveolar basement membrane. On the other hand, AT2 cells are more resistant to injury. They can proliferate, migrate, and diffuse across the basement membrane before differentiating into AT1 cells. AT1 cells have a high level of EMP2 expression [82, 83]. Recent research suggested that EMP2 might be a target in differentiating AT2 cells to AT1 cells, which is regulated by Wnt signaling. The mRNA levels of EMP2 and ITGAV are significantly increased during the AT2-to-AT1 differentiation process. Elevated EMP2 levels have been associated with cancer progression by regulating and administering cell membrane composition and angiogenesis [84, 85]. Therefore, EMP2 increase can promote capillaries formation during the physiological process of AT2-to-AT1 differentiation. ITGAV usually plays an important role in regulating tumor growth and metastasis. However, increased ITGAV expression improves cell adhesion and inhibits lipid transport, which is required for surfactant formation. The increase of ITGAV can block AT2 marker surfactant protein C (SPC) production in the AT1 differentiation process. Since EMP2 can selectively interact with specific integrin subtypes and play an important role in regulating the formation of integrins and lipid rafts on the cell membrane, it is speculated that the EMP2 and ITGAV complex contributes to AT1 differentiation by blocking surfactant production [86].

On AT1 cells, EMP2 guides neutrophil transfer to the alveolar cavity through the epithelium, to regulate innate immunity. The lung host defense is inseparable from successfully migrating circulatory neutrophils (PMNs) to the airway. The transport of PMNs to the lung significantly differs from other tissues [87]. The adhesion and permeability of endothelial cells in alveolar capillaries are integrin-dependent (such as α4β1, α6β1), and PMNs might track along interstitial fibroblasts and finally pass by epithelial cells at the junction of AT1 and AT2 cells [88]. Through homologous interactions with PMNs, epithelial membrane proteins can regulate transepithelial cell migration (TEM) [89]. However, few members of the epithelial membrane protein family have been considered to regulate lung TEM [89–91].

Although the action mechanisms are unclear, EMP2 might promote the recruitment of selected integrins (such as αvβ3, α6β1, α5β1), adhesion molecules, and signaling proteins to lipid rafts on the plasma membrane and downregulate caveolin [78, 85, 92, 93]. Thus, EMP2 can act as a platform for integrin signal transduction in cancer cells and help cell adhesion to the extracellular matrix (ECM). EMP2 is highly expressed in AT1 cells, but absent in AT2 cells. In *Emp2*^*−/−*^ mice, the loss of *Emp2* can lead to the decrease of epithelial raft abundance and an imbalance in the expression of multiple adhesion molecules on the surface of AT1 cells, resulting in the defect of neutrophil migration from epithelial to the alveolar lumen.

In summary, EMP2 is essential in regulating the last step of PMNs transport to infected airspace. In some cases, it has a crucial cost for the integrity of the epithelial barrier and the host's survival.

#### Biological function of EMP2 in cancer

Existing research has suggested that EMP2 can regulate tumor cell proliferation, apoptosis, and metastasis and has vast potential for use in diagnosing and treating some gynecological tumors [94]. Thus, EMP2 might become a new target for tumor diagnosis and treatment. There are different levels of expression in tumor cells. In lung cancer and nasopharyngeal carcinoma cells, only a low level of EMP2expression is detected, which can impact lung cancer cell metastasis and nasopharyngeal carcinoma cell death, indicating that EMP2 might present a tumor suppressor gene role in these two tumors. In breast cancer, ovarian cancer, endometrial cancer, and other tumors, EMP2 levels are usually high, and the higher the expression level of EMP2 in these tumors, the stronger the invasion and metastasis ability of the tumor. Hence, EMP2 expression might have different regulatory mechanisms in diverse tumors, and different expression levels might play different regulatory roles on cells.

Recent studies have shown that EMP2 acts as an oncogene in hormone-related cancers. EMP2 minimally expressed in normal mammary tissue but is highly upregulated in most invasive or triple-negative breast cancer tumors. In human breast cancer cells, the anti-EMP2 antibody significantly inhibits the EMP2-mediated signal transduction and tumor invasion, and promote cell apoptosis, reducing tumor loading in human xenograft and murine metastatic models [95]. Previous research has found that EMP2 might alter integrin (such as αvβ3, α6β1, α5β1) subcellular localization and related signal pathway activity and upregulate FAK and Src phosphorylation, whereas anti-EMP2 treatment can inhibit these reactions. In the past 20 years, the application of monoclonal antibodies in cancer treatment has achieved extensive success. Regarding breast cancer, trastuzumab changed the therapeutic effects of breast cancer patients with HER2/neu overexpression and is one of the few drug options approved by the US Food and Drug Administration (FDA) for women with metastatic breast cancer. Existing evidence suggests that anti-EMP2 treatment might be effective for EMP2-dependent breast cancer. Although anti-EMP2 IgG1 can considerably reduce tumor load (50–80%) in many in vivo breast cancer models, similar to all targeted therapies, anti-EMP2 IgG1 might require combination therapy with other drugs. Liquid biopsy contributes to clinical diagnosis and helps tumor detection, prognosis prediction of patients with curable diseases, monitoring of systematic treatment, and patient stratification based on therapeutic targets or drug resistance mechanisms [96]. Early in the formation and growth of primary tumors such as breast and lung cancers, some tumor cells usually called circulating tumor cells (CTCs), are released into the blood, and can be enriched and detected by different techniques according to their physical and biological characteristics. CTC analysis is considered the cornerstone of real-time liquid biopsy and has great significance in the prognosis of various solid tumors, especially among breast cancer patients [97]. In most current clinical trials, CTCs are detected by testing epithelial markers (such as EpCAM) and cytokeratin peripheral mesenchymal blood cells do not express. However, some epithelial tumor cells display epithelial-to-mesenchymal transition (EMT) characteristics, resulting in reduced epithelial marker expression, increased plasticity and migration and invasion ability, and resistance to anoikis, all necessary markers for CTC survival and transmission [98]. CTC can be detected in the peripheral blood of 40–80% of metastatic breast cancer patients, as well as in the peripheral blood of 10–15% of early breast cancer patients. However, since the EMT is a critical early event in the invasion and metastasis of breast cancer, many breast cancer patients lack EpCAM + CTC, which impairs CTC separation. More study is urgently needed to identify other related biomarkers for CTC identification in breast cancer patients, and to assess the role of novel CTC biomarkers in breast cancer prediction [99]. Many studies have shown that EMP2 is highly expressed in malignancies originating from the epithelium and promotes tumorigenesis. EMP2 is not only a marker of human breast cancer stem cells but also a predictor of adverse clinical results. To evaluate whether EMP2 is a better target for distinguishing breast cancer cells, some studies on breast cancer cell lines used an anti-EMP2 monoclonal antibody to capture MCF-7 and MDA-MB231 breast cancer cells. The results revealed that the recovered component of EMP2 included over three times more MCF-7 cells than the component captured by EpCAM. Also, the anti-EMP2 monoclonal antibody captured over 80% of the total MDA-MB231 cells. EMP2 might be a more efficient biomarker for capturing CTC in breast cancer blood samples and is worthy of further assessments in clinical trials [100].

Recent research has found that tissue-specific human EMP2 is highly expressed in lung tissue but not in lung cancer tissues [4, 101]. The expression levels and location of EMP2 on the plasma membrane can affect several molecules on the membrane, including integrins, CD54 immunoglobulin subfamily members, and GPI-anchored proteins (GPI-AP). EMP2 can also physically regulate the activity of the integrin-FAK signal pathway [92, 102, 103]. However, there is still a short functional study on the effects and mechanisms of EMP2 in lung cancer. Only a few studies have shown that the low EMP2 expression might be closely associated with the poor prognosis and malignant metastasis of lung cancer.

Cell adhesiveness is very important for the metastasis of lung cancer cells. The adhesiveness is primarily determined by keratin phosphorylation and recombination [104–106]. Serine phosphorylation of serine at the 431 positions of keratin 8 (K8) can promote the recombination of K8 around the nucleus. Sphingosine phosphocholine (SPC) can promote the recombination of K8 by activating JNK, ERK, phosphorylating K8, and protein phosphatase 2A (PP2A) [107–110]. In A549 and H1299 lung cancer cells, EMP2 downregulation by SPC can specifically promote caveolin-1 expression, enhance the combination of caveolin-1 and phosphatase 2A (protein phosphatase2a, PP2A), and induce PP2A detachment from its binding protein alpha 4, and finally result in PP2A ubiquitination and degradation. Without alpha 4 binding, the catalytic subunit of PP2A rapidly declines, and the dephosphorylation of PP2A family substrates is impaired, which will promote JNK and ERK phosphorylation and induce K8 phosphorylation and recombination, indirectly affecting the adhesion of tumor cells, and promoting tumor deterioration [111]. Therefore, low EMP2 levels in lung cancer might be an essential factor in the physical properties of lung cancer cells' migration and invasion. Furthermore, EMP2 expression is linked to the formation of various tumors, suggesting that it might be a potential novel molecular diagnostic marker. The expression of EMP2 in endometrial cancer patients' tissues positively correlates with endometrial cancer malignancy [112]. Endometrial cancer with high EMP2 expression has stronger myometrial invasiveness, higher clinical stage, stronger recurrence probability, and mortality, and it is more challenging to treat [113]. Other studies have shown that EMP2 is co-expressed with tumor stem cell-related marker aldehyde dehydrogenase 1 (ALDH1) in endometrial cancer. EMP2 can promote ALDH1 expression and activation and be used as a specific molecular diagnostic marker for CSC identification [114]. In an ovarian cancer transplanted tumor model, anti EMP2 divalent antibody therapy reduces ovarian cancer cell proliferation and promotes apoptosis in vivo [115]. In malignant glioma samples, the proportion of high EMP2 expression is significantly higher than in low-grade glioma. Also, EMP2 can promote abnormal angiogenesis of malignant glioma by regulating the expression of VEGF-A and angiogenesis-related STAT3 cell signaling pathway [116–118]. In U373, U87MG, and gm97 malignant glioma cells, the expression of integrin αVβ3 on the cell surface is also positively correlated with EMP2, upregulating its expression, increasing the expression of integrin αVβ3, and promoting the proliferation and invasion of cells through activation of the FAK/SRC downstream pathway [103]. In nasopharyngeal carcinoma, EMP2 can exist as a tumor suppressor. EMP2 can induce apoptosis in the CNE-2 nasopharyngeal carcinoma cell line and expose cells to radiotherapy [119]. The lncRNA nuclear enriched abundant transcript 1 (NEAT1) can affect EMP2 expression via miR-101-3p and slow the progression of nasopharyngeal carcinoma [120]. EMP2 might also be a tumor suppressor in bladder urothelial carcinoma (BLCA). EMP2 can induce G2/M phase arrest of the BLCA cell line and inhibit cell proliferation and clone formation. A study based on chromosome immunoprecipitation technology also found that the *EMP2* transcription and translation in BLCA cells can be promoted by the transcription factor cAMP-responsive element-binding protein 1 (CREB1) [121].

The differential expression of EMP2 in various tumor tissues plays different roles. A complete study of its mechanism would help research and develop new tumor treatment targets and methods. It can reduce tumor cell proliferation, induce apoptosis, and inhibit metastasis by regulating EMP2 expression in tumor cells. Using EMP2 to identify endometrial cancer and CTCs demonstrated its potential as a tumor molecular diagnostic marker. Current EMP2 research is inadequately comprehensive. The regulation of gene expression and specific signal pathways of EMP2 in different tumor cells must be further studied to develop more effective tumor diagnosis and treatment methods.

### EMP3

EMP3 is also expressed in many tissues and organs. The mRNA level of EMP3 are higher in adult peripheral blood leukocytes and lower in the brain [1]. It is a functional protein that regulates cell proliferation, and differentiation, activating the caspase-dependent apoptosis pathway and cell–cell interaction [8]. EMP3 bears hypermethylation mediated transcriptional silencing in both glioma and neuroblastoma 19q13 chromosome regions, it was initially considered a tumor suppressor gene in glioma and neuroblastoma [122]. Oligodendroglioma patients with a heterozygous deletion of the *EMP3* gene on chromosome 19 have a higher recurrence rate and mortality. In neuroblastoma, the *EMP3* gene is hypermethylated and silenced. Demethylating agents can restore the *EMP3* expression in neuroblastoma cells, significantly decreasing cell proliferation. In neuroblastoma tumors, the hypermethylation of the *EMP3* gene, which leads to the EMP3 downregulation, is also closely related to the low survival rate of patients [123]. CD44 is a transmembrane adhesion glycoprotein and was indicated to be a promising predictor for poor prognosis in glioblastoma multiforme (GBM). [124]. In GBM with high CD44 expression, EMP3 is frequently up-regulated and might be a vital role in tumor initiation and advancement in primary GBM. The molecular mechanism involved in the promoting of CD44-high GBM by EMP3 may mainly for the reason that EMP3 could regulate the activation of transforming growth factor-β (TGF-β) [125]. The TGF-β signaling pathway was known for the intricate role in regulation of multiple cellular physiological process including cell proliferation, apoptosis, invasion, immune responses. TGF-β pathway might serve as tumor-suppressor in early stages of tumor initiation while TGF-β also could elicit tumor promoting effects in certain cancer cells including glioma cells [126]. Recently studies found that EMP3 can interact with the TGF-β receptor and activate the TGF-β/Smad2/3 signaling pathway. Also, EMP3 deletion can induce the expression of apoptosis markers [125]. The latest glioblastoma reports, combined with bioinformatics research and in vivo and in vitro investigations, confirmed that EMP3 was also related to GBM immunosuppression [127]. Programmed death-ligand 1 (PD-L1) and programmed cell death-1 (PD-1) are a pair of proteins that are critical for maintaining immune homeostasis. PD-L1 overexpressed on some cancer cells could bind to the PD-1 on immune cells, such as the tumor-infiltrating lymphocytes (TILs), leading to in impaired T cell activation and cancer immune escape [128]. Tumor-associated macrophages (TAMs) are also a kind of pivotal components in the tumor microenvironment and could influence the development of tumor including tumor metastasis, angiogenesis and immunosuppression [129]. There are some evidences that the high EMP3 expression in GBM is accompanied by high PD-L1 expression and infiltration of tumor-associated macrophages (TAM). Besides, the infiltration of CD4 + and CD8 + T cells was inhibited by inhibiting the secretion of CXCL9 and CXCL10 by macrophages [127].

Evidence has shown that EMP3 is a candidate tumor suppressor gene for some solid tumors, such as nervous system tumors, which can significantly inhibit the proliferation of tumor cells. Thus, EMP3 act as a tumor suppressor gene. Various studies have reached different conclusions on the role of EMP3 in breast cancer. According to much research, EMP3 promotes breast cancer progression and is highly expressed in breast cancer. Additionally, miR-765 can regulate EMP3 in some primary breast cancer tissues [130]. Recent studies have also shown that EMP3 is associated with a good survival rate during breast cancer progression, negatively regulates the S phase of the breast cancer cell cycle, inhibits BRCA1 and RAD51 expression after repair of DNA double-strand breaks, interferes with DNA replication and damage repair and stem cell-like characteristics of breast cancer cells, blocks Akt-mTOR signal activation of autophagy, and enhance the sensitivity of breast cells to adriamycin [131].

In biliary malignant gallbladder carcinoma (GBC), low EMP3 expression is associated with a poor prognosis of GBC. EMP3 knockout can promote the proliferation of cancer cells, while overexpression impairs the migration ability of cells and promotes apoptosis. In GBC cells, miR-663a is overexpressed, downregulates EMP3, and further activates the MAPK/ERK signaling pathway to regulate GBC progression [132]. Nevertheless, in urothelial and hepatocellular carcinomas, EMP3 shows more potential cancer-promoting effects. In urothelial cell carcinoma, EMP3 can enhance the proliferation and migration of cancer cells and is related to the activation of the ErbB2-PI3K-AKT pathway, resulting in enhanced signal transduction of the FAK/SRC pathway, activation of RhoA and Rock1/2 and up-regulation of ɑ1, ɑ2, ɑ3, ɑ5,ɑV, ɑ6 and β1 integrins in vitro [133]. In oral squamous cell carcinoma, miR-765 might be involved in regulating EMP3. The reduction of EMP3 can reduce the ability of oral squamous cell carcinoma cells to migrate [134]. High EMP3 expression is associated with a worse overall survival rate [Hazard ratio (HR) = 1.71; 95% confidence interval (CI) = 1.32–2.22; *p* < 0.0001] and progression-free survival in gastric cancer (HR = 1.99; 95% CI = 1.45–2.72; *p* < 0.0001). In gastric cancer cells, EMP3 can act as a downstream effector of TWIST1 and TWIST2 and participate in the EMT of gastric cancer [135]. Recent research has shown EMP3 is a positive regulatory fulcrum in reversing GC cell resistance to cisplatin in gastric cancer treatment. In GC cells, the up-regulation of the transmembrane glycoprotein CD13 inhibits the hypermethylation of the CpG island of the *EMP3* gene, leading to the cisplatin (CDDP) resistance of GC cells. Ubenimex can down-regulate EMP3 and weaken the activation of the CD13/EMP3/PI3K/Akt/NF-κB pathway, reversing the CDDP resistance in GC. In this resistance, autophagy is inhibited, followed by increased CDDP-induced apoptosis, EMT development is hindered, and cell migration and invasion are weakened [136].

The role of EMP3 in lung cancer has not been fully clarified and the molecular mechanism in non-small cell lung cancer need to be confirmed further. Some studies have shown that EMP3 is much lower in non-small cell lung cancer than in normal control tissue, which is connected to the TMN stage, but there is no significant correlation with other clinical parameters [137]. However, some other studies have shown that EMP3 mRNA is only inhibited in a small minority of lung cancer cell lines, and most of the lung cancer cell lines under test are highly expressed [138], which is positively correlated with the low survival rate of patients, and indicating that EMP3 may perform tumor promoting function. ALDH1 promotes the up-regulation of EMP3 in NSCLC cells and participates in the regulation of cell proliferation, anti radiation ability of cancer stem cells (CSCs) and epithelial mesenchymal transition (EMT) of NSCLC cells. In lung CSC (LCSC), EMP3 could activate TGF‑β / Smad signal pathway by binding with TGF‑β receptor type 2 (TGFBR2), results in the effect of CSCs and EMT [139]. EMP3 is significantly expressed in hepatocellular carcinoma, promoting the invasion and migration of hepatocellular carcinoma cells. Also, EMP3 knockout can result in G1 phase arrest. The biological role of EMP3 is still mostly unknown. Studies have been conducted on its role in controlling cell proliferation and death based on its structural homology with the same family of proteins. These results showed that the changes in EMP3 expression have different effects on cell signaling pathways, cell proliferation activity, and behavior.

## Conclusion and future

Herein, we reviewed the structure, gene mutation sites, post-transcriptional modification sites, and functions of four members of the PMP22 family in different tissues, especially in different tumor tissues. PMP22 family members are present in various levels of expression imbalance in various cancers. In some instances, they act as tumor suppressors, whereas in others, they act as oncoproteins, promoting cancer progression and metastasis. These differences might be related to the difference in gene mutation of PMP22 family members in diverse tissues and the results of various post-transcriptional regulations. In recent decades, increasing studies have provided evidence for the mechanisms of PMP22 family members in malignant tumors. Nevertheless, the molecular mechanism mediated by PMP22 is quite complex, and whether gene mutations and different post transcriptional modifications in PMP22 family members play a critical role also urgently needs further in-depth research. The study of the biological roles of PMP22 family members might lead to promising targets for the early diagnosis, prognosis, and treatment of human cancers.

## Supplementary Information

**Additional file 1: Table S1.** Missense substitution of epithelial membrane protein family members.

## Data Availability

Not applicable.

## Abbreviations

PMP22: Peripheral myelin protein 22
EMP2: Epithelial membrane proteins
TSGs: Tumor suppressor genes
GAS3: Growth arrest-specific 3
PNS: Peripheral nervous system
SNV: Single nucleotide variation
SAV: Single amino acid variation
PTMs: Post-Translational modifications
GlcNAc: N-Acetylglucosamine
Gal: Galactose
Neu5Ac: N-Acetylneuraminic acid
GalN: Galactosamine
CK2: Casein kinase 2
SAM: S-Adenosylmethionine
Smurf1: Ubiquitin regulator 1
TMP: Tumor-associated membrane protein
CMT: Charcot Marie Tooth disease
HNPP: Hereditary neuropathy with liability to pressure palsies
BrdU: 5-Bromo-2'-Deoxy-Uridine
ZO-1: Tight junction protein 1
SCs: Schwann cells
Arf-6: ADP ribosylation factor 6
TGF-β2: Transforming growth factor β2
TGFBR2: TGFβ receptor type 2
EMT: Epithelial-mesenchymal transformation
BLCA: Bladder urothelial carcinoma
TFF3: Trefoil factor family peptide 3
miRNA: MicroRNA
DDP: Cisplatin
Yap: Yes associated protein 1
TAZ: Transcriptional coactivator with PDZ binding motif
ARPE-19: Retinal pigment epithelium
PVR: Vitreous body retinopathy
GEO: Gene expression omnibus
AT1: Type 1 alveolar epithelial cells
SPC: Surfactant protein C
PMNs: Polymorphonuclearneutrophil
TEM: Transepithelial cell migration
ECM: Extracellular matrix
FDA: US Food and Drug Administration
CTC: Circulating tumor cells
GPI-AP: GPI-Anchored proteins
K8: Keratin 8
SPC: Sphingosine phosphocholine
PP2A: Protein phosphatase2a
ALDH1: Aldehyde dehydrogenase 1
NEAT1: Nuclear enriched abundant transcript 1
CREB1: Camp-responsive element-binding protein 1
GBM: Glioblastoma multiforme
TAM: Tumor-associated macrophages
CSCs: Cancer stem cells
