# Correction: Unraveling the structures, functions and mechanisms of epithelial membrane protein family in human cancers

**DOI:** 10.1186/s40164-022-00340-8

**Published:** 2022-11-08

**Authors:** Nan Zhang, Hong‑Ping Zhu, Wei Huang, Xiang Wen, Xin Xie, Xian Jiang, Cheng Peng, Bo Han, Gu He

**Affiliations:** 1 State Key Laboratory of Southwestern Chinese Medicine Resources, School of Pharmacy, Hospital of Chengdu University of Traditional Chinese Medicine, Chengdu University of Traditional Chinese Medicine, Chengdu, 611137 China; 2 Department of Dermatology, West China Hospital, Sichuan University, Chengdu, 610041 Sichuan China; 3 Laboratory of Dermatology, Clinical Institute of Inflammation and Immunology (CIII), Frontiers Science Center for Disease-Related Molecular Network and State Key Laboratory of Biotherapy, West China Hospital, Sichuan University and Collaborative Innovation Center of Biotherapy, Chengdu, 610041 China; 4 Antibiotics Research and Re‑Evaluation Key Laboratory of Sichuan Province, Sichuan Industrial Institute of Antibiotics, Chengdu University, Chengdu, 610106 China

## Correction: Experimental Hematology & Oncology (2022) 11:69 https://doi.org/10.1186/s40164-022-00321-x

In this article [1] the wrong figure appeared as Fig. 3, this is a duplication of Fig. 2. Figure 3 in the original version of this article has been replaced with correct figure. The figure should have appeared as shown in this Correction.

**Fig. 3:**
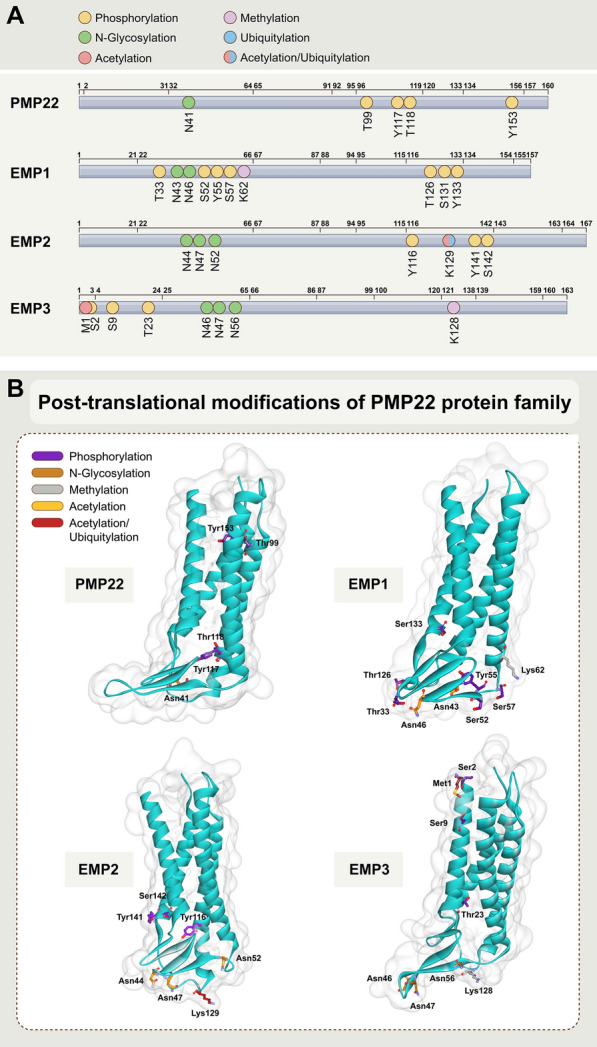
Post-translational modifications of PMP22 protein family. **A** Schematic view of structure of the PMP22 family of proteins with post-translational modification sites. The upper numbers refer to the starting amino acid sites of different domains, and the lower dotted line refers to the mutated amino acid sites. **B** Post-translational modification sites of PMP22 protein family. The protein structures were modelled by AlphaFold2 [13]
