# Supplementary material for: Unraveling the structures, functions and mechanisms of epithelial membrane protein family in human cancers
Source: Exp Hematol Oncol. 2022 Oct 10;11:69. doi: 10.1186/s40164-022-00321-x (PMC9552464; doi:10.1186/s40164-022-00321-x)
Supplement: Supplementary file 1 — Additional file 1: Table S1. Missense substitution of epithelial membrane protein family members. [file 40164_2022_321_MOESM1_ESM.docx]

**Table S1. Missense substitution of epithelial membrane protein family members.**

| **Gene Name** | **Transcript** | **Census Tier 1** | **Sample Name** | **Sample ID** | **AA Mutation** | **CDS Mutation** | **Primary Tissue** | **Tissue Subtype 1** | **Tissue Subtype 2** | | | | | | | | **Zygosity** | **Genomic Co-ordinates** |
| --- | --- | --- | --- | --- | --- | --- | --- | --- | --- | --- | --- | --- | --- | --- | --- | --- | --- | --- |
| EMP1 | ENST00000256951.9 | No | LICA-CN-HCC052T | 2634099 | p.L2F | c.6G>T | Liver | NS | NS | Other | Neoplasm | NS | - | 660 | Confirmed Somatic | Tumour Sample | Unknown | 12:13211516..13211516 |
| EMP1 | ENST00000256951.9 | No | LICA-CN-HCC052T | 2634099 | p.V3L | c.7G>T | Liver | NS | NS | Other | Neoplasm | NS | - | 660 | Confirmed Somatic | Tumour Sample | Unknown | 12:13211517..13211517 |
| EMP1 | ENST00000256951.9 | No | CAL27 | 2296292 | p.V11G | c.32T>G | Upper aerodigestive tract | Head neck | NS | Carcinoma | Squamous cell carcinoma | NS | 25275298 | - | Previously Reported | Cultured | Unknown | 12:13211542..13211542 |
| EMP1 | ENST00000256951.9 | No | NCI-H322M | 1998462 | p.V11G | c.32T>G | Lung | NS | NS | Carcinoma | Bronchioloalveolar adenocarcinoma | NS | 23856246 | - | Confirmed Somatic | Cultured | Homozygous | 12:13211542..13211542 |
| EMP1 | ENST00000256951.9 | No | SK-MEL-5 | 1998477 | p.V11G | c.32T>G | Skin | Axilla | NS | Malignant melanoma | NS | NS | 23856246 | - | Confirmed Somatic | Cultured | Heterozygous | 12:13211542..13211542 |
| EMP1 | ENST00000256951.9 | No | IM-95m | 2807628 | p.V16L | c.46G>C | Stomach | NS | NS | Carcinoma | Adenocarcinoma | NS | 24807215 | - | Variant of unknown origin | Cultured | Unknown | 12:13211556..13211556 |
| EMP1 | ENST00000256951.9 | No | TCGA-DU-6404-02 | 2185814 | p.A25G | c.74C>G | Central nervous system | Brain | NS | Glioma | NS | NS | - | 545 | Confirmed Somatic | Unknown | Unknown | 12:13211584..13211584 |
| EMP1 | ENST00000256951.9 | No | BD114T | 2459740 | p.V27I | c.79G>A | Biliary tract | Bile duct | NS | Carcinoma | NS | NS | - | 658 | Confirmed Somatic | Tumour Sample | Unknown | 12:13213479..13213479 |
| EMP1 | ENST00000256951.9 | No | TCGA-DD-AAW0-01 | 2340624 | p.S31Y | c.92C>A | Liver | NS | NS | Carcinoma | Hepatocellular carcinoma | NS | - | 628 | Confirmed Somatic | Tumour Sample | Unknown | 12:13213492..13213492 |
| EMP1 | ENST00000256951.9 | No | TCGA-BR-A4PE-01 | 2197892 | p.T33M | c.98C>T | Stomach | NS | NS | Carcinoma | Adenocarcinoma | NS | - | 541 | Confirmed Somatic | Unknown | Unknown | 12:13213498..13213498 |
| EMP1 | ENST00000256951.9 | No | TCGA-OL-A6VO-01 | 2339991 | p.T33M | c.98C>T | Breast | NS | NS | Carcinoma | NS | NS | - | 414 | Confirmed Somatic | Tumour Sample | Unknown | 12:13213498..13213498 |
| EMP1 | ENST00000256951.9 | No | V-PH-03T | 2329059 | p.C44F | c.131G>T | Adrenal gland | Adrenal gland | NS | Pheochromocytoma | Malignant | NS | 25545346 | - | Confirmed Somatic | Tumour Sample | Heterozygous | 12:13213531..13213531 |
| EMP1 | ENST00000256951.9 | No | TCGA-E2-A1L7-01 | 1900172 | p.T45A | c.133A>G | Breast | NS | NS | Carcinoma | NS | NS | - | 414 | Confirmed Somatic | Tumour Sample | Unknown | 12:13213533..13213533 |
| EMP1 | ENST00000256951.9 | No | SJOS001118_D1 | 2372227 | p.I47T | c.140T>C | Bone | Femur | NS | Osteosarcoma | NS | NS | 24703847 | - | Confirmed Somatic | Unknown | Unknown | 12:13213540..13213540 |
| EMP1 | ENST00000256951.9 | No | 5-2A | 2746100 | p.S50N | c.149G>A | Large intestine | NS | NS | Carcinoma | Adenocarcinoma | NS | - | 646 | Confirmed Somatic | Tumour Sample | Unknown | 12:13213549..13213549 |
| EMP1 | ENST00000256951.9 | No | TCGA-AM-5820-01 | 1651150 | p.S57N | c.170G>A | Large intestine | Colon | Sigmoid | Carcinoma | Adenocarcinoma | NS | - | 376 | Confirmed Somatic | Unknown | Unknown | 12:13213570..13213570 |
| EMP1 | ENST00000256951.9 | No | 587256 | 1766760 | p.D59Y | c.175G>T | Large intestine | Colon | NS | Carcinoma | Adenocarcinoma | NS | 22895193 | - | Previously Reported | Tumour Sample | Unknown | 12:13213575..13213575 |
| EMP1 | ENST00000256951.9 | No | TCGA-EE-A3AA-06 | 2121692 | p.D59Y | c.175G>T | Skin | NS | NS | Malignant melanoma | NS | NS | - | 540 | Confirmed Somatic | Unknown | Unknown | 12:13213575..13213575 |
| EMP1 | ENST00000256951.9 | No | T3235 | 2658250 | p.K62N | c.186G>T | Large intestine | NS | NS | Carcinoma | Adenocarcinoma | NS | 27149842 | - | Confirmed Somatic | Unknown | Unknown | 12:13213691..13213691 |
| EMP1 | ENST00000256951.9 | No | TCGA-IR-A3LH-01 | 2193368 | p.F67L | c.201C>G | Cervix | NS | NS | Carcinoma | Squamous cell carcinoma | NS | - | 415 | Confirmed Somatic | Unknown | Unknown | 12:13213706..13213706 |
| EMP1 | ENST00000256951.9 | No | 11 | 2557193 | p.M68T | c.203T>C | Upper aerodigestive tract | Mouth | NS | Carcinoma | Squamous cell carcinoma | NS | 26934577 | - | Variant of unknown origin | Tumour Sample | Unknown | 12:13213708..13213708 |
| EMP1 | ENST00000256951.9 | No | TCGA-A3-A6NL-01 | 2339286 | p.V81L | c.241G>C | Kidney | NS | NS | Carcinoma | Clear cell renal cell carcinoma | NS | - | 416 | Confirmed Somatic | Tumour Sample | Unknown | 12:13213746..13213746 |
| EMP1 | ENST00000256951.9 | No | TCGA-ER-A19F-06 | 2121717 | p.F87V | c.259T>G | Skin | NS | NS | Malignant melanoma | NS | NS | - | 540 | Confirmed Somatic | Unknown | Unknown | 12:13213764..13213764 |
| EMP1 | ENST00000256951.9 | No | TCGA-IR-A3LH-01 | 2193368 | p.F87L | c.261C>G | Cervix | NS | NS | Carcinoma | Squamous cell carcinoma | NS | - | 415 | Confirmed Somatic | Unknown | Unknown | 12:13213766..13213766 |
| EMP1 | ENST00000256951.9 | No | 94128 | 2807570 | p.R94Q | c.281G>A | Stomach | NS | NS | Carcinoma | Adenocarcinoma | NS | 24807215 | - | Confirmed Somatic | Tumour Sample | Unknown | 12:13213786..13213786 |
| EMP1 | ENST00000256951.9 | No | RK099_C01 | 1918871 | p.L97F | c.289C>T | Liver | NS | NS | Carcinoma | NS | NS | - | 322 | Variant of unknown origin | Tumour Sample | Heterozygous | 12:13213794..13213794 |
| EMP1 | ENST00000256951.9 | No | 117 | 2748089 | p.S98L | c.293C>T | Stomach | NS | NS | Carcinoma | NS | NS | - | 683 | Confirmed Somatic | Tumour Sample | Unknown | 12:13213798..13213798 |
| EMP1 | ENST00000256951.9 | No | T3202 | 2296197 | p.L107P | c.320T>C | Large intestine | Caecum | NS | Carcinoma | Adenocarcinoma | NS | 25344691 | - | Confirmed Somatic | Unknown | Unknown | 12:13214537..13214537 |
| EMP1 | ENST00000256951.9 | No | LICA-CN-HCC064T | 2634111 | p.L107Q | c.320T>A | Liver | NS | NS | Other | Neoplasm | NS | - | 660 | Confirmed Somatic | Tumour Sample | Unknown | 12:13214537..13214537 |
| EMP1 | ENST00000256951.9 | No | T3202 | 2658288 | p.L107P | c.320T>C | Large intestine | NS | NS | Carcinoma | Adenocarcinoma | NS | 27149842 | - | Confirmed Somatic | Unknown | Unknown | 12:13214537..13214537 |
| EMP1 | ENST00000256951.9 | No | APGI-CC-1601 | 2866086 | p.Y116S | c.347A>C | Biliary tract | Bile duct | NS | Carcinoma | Adenocarcinoma | NS | 26804919 | - | Confirmed Somatic | Tumour Sample | Unknown | 12:13214564..13214564 |
| EMP1 | ENST00000256951.9 | No | TCGA-CA-6717-01 | 1651188 | p.Y116C | c.347A>G | Large intestine | Colon | Ascending | Carcinoma | Adenocarcinoma | NS | - | 376 | Confirmed Somatic | Unknown | Unknown | 12:13214564..13214564 |
| EMP1 | ENST00000256951.9 | No | TCGA-EE-A2GC-06 | 2121655 | p.Y120N | c.358T>A | Skin | NS | NS | Malignant melanoma | NS | NS | - | 540 | Confirmed Somatic | Unknown | Unknown | 12:13214575..13214575 |
| EMP1 | ENST00000256951.9 | No | ESO-007 | 1890847 | p.A121V | c.362C>T | Oesophagus | NS | NS | Carcinoma | Adenocarcinoma | NS | 23525077 | - | Variant of unknown origin | Tumour Sample | Unknown | 12:13214579..13214579 |
| EMP1 | ENST00000256951.9 | No | WGC067107 | 2549364 | p.G131S | c.391G>A | Large intestine | NS | NS | Carcinoma | Adenocarcinoma | NS | - | 646 | Confirmed Somatic | Tumour Sample | Unknown | 12:13214608..13214608 |
| EMP1 | ENST00000256951.9 | No | TCGA-RD-A8N6-01 | 2263154 | p.G131S | c.391G>A | Stomach | NS | NS | Carcinoma | Adenocarcinoma | NS | - | 541 | Confirmed Somatic | Tumour Sample | Unknown | 12:13214608..13214608 |
| EMP1 | ENST00000256951.9 | No | TCGA-F5-6814-01 | 1651640 | p.F141L | c.423C>A | Large intestine | Rectum | NS | Carcinoma | Adenocarcinoma | NS | - | 375 | Confirmed Somatic | Tumour Sample | Unknown | 12:13214640..13214640 |
| EMP1 | ENST00000256951.9 | No | STC232 | 2404160 | p.V149I | c.445G>A | Stomach | NS | NS | Carcinoma | Intestinal adenocarcinoma | NS | 25042771 | - | Confirmed Somatic | Tumour Sample | Unknown | 12:13214662..13214662 |
| EMP1 | ENST00000256951.9 | No | TCGA-F7-A624-01 | 2193851 | p.V149I | c.445G>A | Upper aerodigestive tract | Head neck | NS | Carcinoma | Squamous cell carcinoma | NS | - | 627 | Confirmed Somatic | Unknown | Unknown | 12:13214662..13214662 |
| EMP1 | ENST00000256951.9 | No | TCGA-B5-A11E-01 | 1783399 | p.R155I | c.464G>T | Endometrium | NS | NS | Carcinoma | Endometrioid carcinoma | NS | - | 419 | Variant of unknown origin | Tumour Sample | Heterozygous | 12:13214681..13214681 |
| EMP1 | ENST00000256951.9 | No | LUAD-S01362 | 1765144 | p.K156Q | c.466A>C | Lung | NS | NS | Carcinoma | Adenocarcinoma | NS | 22980975 | - | Variant of unknown origin | Tumour Sample | Unknown | 12:13214683..13214683 |
| EMP1 | ENST00000256951.9 | No | TCGA-D1-A16X-01 | 1783518 | p.K156N | c.468G>T | Endometrium | NS | NS | Carcinoma | Endometrioid carcinoma | NS | - | 419 | Variant of unknown origin | Tumour Sample | Heterozygous | 12:13214685..13214685 |
| EMP2 | ENST00000359543.7 | No | MSU3-a | 2809972 | p.K167N | c.501A>T | Large intestine | Colon | Transverse | Adenoma | Tubulovillous | NS | 26336987 | - | Confirmed Somatic | Tumour Sample | Unknown | 16:10532908..10532908 |
| EMP2 | ENST00000359543.7 | No | MSU3-c | 2809980 | p.K167N | c.501A>T | Large intestine | Colon | Transverse | Carcinoma | Adenocarcinoma | NS | 26336987 | - | Confirmed Somatic | Tumour Sample | Unknown | 16:10532908..10532908 |
| EMP2 | ENST00000359543.7 | No | SNU-C4 | 2302011 | p.L163P | c.488T>C | Large intestine | NS | NS | Carcinoma | Adenocarcinoma | NS | 24755471 | - | Variant of unknown origin | Cultured | Heterozygous | 16:10532921..10532921 |
| EMP2 | ENST00000359543.7 | No | H920 | 2776291 | p.L161P | c.482T>C | Lung | NS | NS | Carcinoma | Adenocarcinoma | NS | 29681454 | - | Variant of unknown origin | Unknown | Unknown | 16:10532927..10532927 |
| EMP2 | ENST00000359543.7 | No | CHC1754T | 2340455 | p.G157V | c.470G>T | Liver | NS | NS | Carcinoma | Hepatocellular carcinoma | NS | - | 381 | Confirmed Somatic | Tumour Sample | Unknown | 16:10532939..10532939 |
| EMP2 | ENST00000359543.7 | No | TCGA-ER-A2ND-06 | 2121732 | p.T153I | c.458C>T | Skin | NS | NS | Malignant melanoma | NS | NS | - | 540 | Confirmed Somatic | Unknown | Unknown | 16:10532951..10532951 |
| EMP2 | ENST00000359543.7 | No | 5-VS037-T7 | 2869867 | p.W147L | c.440G>T | Skin | NS | NS | Carcinoma | Basal cell carcinoma | NS | 26950094 | - | Confirmed Somatic | Tumour Sample | Unknown | 16:10532969..10532969 |
| EMP2 | ENST00000359543.7 | No | TCGA-DX-AB2W-01 | 2457277 | p.A146E | c.437C>A | Soft tissue | NS | NS | Sarcoma | NS | NS | - | 635 | Confirmed Somatic | Tumour Sample | Unknown | 16:10532972..10532972 |
| EMP2 | ENST00000359543.7 | No | TARGET-50-PAJMLZ-01A-01D | 2459483 | p.L145R | c.434T>G | Kidney | NS | NS | Wilms tumour | NS | NS | - | 657 | Confirmed Somatic | Tumour Sample | Unknown | 16:10532975..10532975 |
| EMP2 | ENST00000359543.7 | No | PT25 | 2521282 | p.Y143C | c.428A>G | Skin | NS | NS | Carcinoma | Basal cell carcinoma | NS | 25759019 | - | Confirmed Somatic | Tumour Sample | Unknown | 16:10532981..10532981 |
| EMP2 | ENST00000359543.7 | No | T3641 | 2658286 | p.Y141H | c.421T>C | Large intestine | NS | NS | Carcinoma | Adenocarcinoma | NS | 27149842 | - | Confirmed Somatic | Unknown | Unknown | 16:10532988..10532988 |
| EMP2 | ENST00000359543.7 | No | PD42114a | 2894823 | p.G140S | c.418G>A | Skin | Head neck | NS | Malignant melanoma | Superficial spreading | NS | 33024263 | - | Confirmed Somatic | Tumour Sample | Unknown | 16:10532991..10532991 |
| EMP2 | ENST00000359543.7 | No | HKNPC-001-Tumor-SM-5ZSUO | 2640098 | p.G140S | c.418G>A | Upper aerodigestive tract | Pharynx | Nasopharynx | Carcinoma | Nasopharyngeal carcinoma | NS | 28098136 | - | Confirmed Somatic | Tumour Sample | Unknown | 16:10532991..10532991 |
| EMP2 | ENST00000359543.7 | No | TCGA-AX-A0J1-01 | 1783377 | p.G140S | c.418G>A | Endometrium | NS | NS | Carcinoma | Endometrioid carcinoma | NS | - | 419 | Previously Reported | Tumour Sample | Heterozygous | 16:10532991..10532991 |
| EMP2 | ENST00000359543.7 | No | J54 | 2815393 | p.V133M | c.397G>A | Central nervous system | Brain | NS | Glioma | Astrocytoma Grade IV | Glioblastoma multiforme | 29263181 | - | Confirmed Somatic | Tumour Sample | Unknown | 16:10533012..10533012 |
| EMP2 | ENST00000359543.7 | No | CSCC-16-T | 2292464 | p.P132L | c.395C>T | Skin | Head neck | NS | Carcinoma | Squamous cell carcinoma | NS | 25303977 | - | Confirmed Somatic | Tumour Sample | Heterozygous | 16:10533014..10533014 |
| EMP2 | ENST00000359543.7 | No | TCGA-ER-A42L-06 | 2456271 | p.P132L | c.395C>T | Skin | NS | NS | Malignant melanoma | NS | NS | - | 540 | Confirmed Somatic | Tumour Sample | Unknown | 16:10533014..10533014 |
| EMP2 | ENST00000359543.7 | No | GCTK_480_T | 2747480 | p.A128T | c.382G>A | Stomach | NS | NS | Carcinoma | NS | NS | - | 683 | Confirmed Somatic | Tumour Sample | Unknown | 16:10533027..10533027 |
| EMP2 | ENST00000359543.7 | No | TARGET-10-PAKSWW-09A-01D | 2177230 | p.A128T | c.382G>A | Haematopoietic and lymphoid | NS | NS | Haematopoietic neoplasm | Acute lymphoblastic leukaemia | NS | - | 533 | Confirmed Somatic | Unknown | Unknown | 16:10533027..10533027 |
| EMP2 | ENST00000359543.7 | No | TCGA-BK-A56F-01 | 2198448 | p.A128T | c.382G>A | Endometrium | NS | NS | Carcinoma | Endometrioid carcinoma | NS | - | 419 | Confirmed Somatic | Unknown | Unknown | 16:10533027..10533027 |
| EMP2 | ENST00000359543.7 | No | TCGA-14-0867-01 | 2178106 | p.E121K | c.361G>A | Central nervous system | Brain | NS | Glioma | Astrocytoma Grade IV | Glioblastoma multiforme | - | 329 | Confirmed Somatic | Unknown | Unknown | 16:10533048..10533048 |
| EMP2 | ENST00000359543.7 | No | SJALL043847-R | 2824112 | p.R120H | c.359G>A | Haematopoietic and lymphoid | NS | NS | Lymphoid neoplasm | Acute lymphoblastic B cell leukaemia | NS | 31697823 | - | Confirmed Somatic | Tumour Sample | Unknown | 16:10533050..10533050 |
| EMP2 | ENST00000359543.7 | No | TCGA-AA-3713-01 | 1651032 | p.R120H | c.359G>A | Large intestine | Colon | Ascending | Carcinoma | Adenocarcinoma | NS | - | 376 | Confirmed Somatic | Unknown | Unknown | 16:10533050..10533050 |
| EMP2 | ENST00000359543.7 | No | N-Thy004 | 2406673 | p.R119S | c.357G>T | Thymus | NS | NS | Thymic carcinoma | Undifferentiated carcinoma | NS | 24974848 | - | Confirmed Somatic | Tumour Sample | Unknown | 16:10533052..10533052 |
| EMP2 | ENST00000359543.7 | No | TCGA-78-7156-01 | 2194850 | p.Y116F | c.347A>T | Lung | NS | NS | Carcinoma | Adenocarcinoma | NS | - | 417 | Confirmed Somatic | Unknown | Unknown | 16:10533062..10533062 |
| EMP2 | ENST00000359543.7 | No | PD4203a | 1351069 | p.S114Y | c.341C>A | NS | NS | NS | NS | NS | NS | 22722201 | 385 | Confirmed Somatic | Tumour Sample | Unknown | 16:10533068..10533068 |
| EMP2 | ENST00000359543.7 | No | TCGA-XF-A9SK-01 | 2457406 | p.S114C | c.341C>G | Urinary tract | Bladder | NS | Carcinoma | NS | NS | - | 413 | Confirmed Somatic | Tumour Sample | Unknown | 16:10533068..10533068 |
| EMP2 | ENST00000359543.7 | No | TCGA-77-8143-01 | 2195219 | p.V109I | c.325G>A | Lung | NS | NS | Carcinoma | Squamous cell carcinoma | NS | - | 418 | Confirmed Somatic | Unknown | Unknown | 16:10533084..10533084 |
| EMP2 | ENST00000359543.7 | No | TCGA-DD-A115-01 | 2194611 | p.C108R | c.322T>C | Liver | NS | NS | Carcinoma | Hepatocellular carcinoma | NS | - | 628 | Confirmed Somatic | Unknown | Unknown | 16:10533087..10533087 |
| EMP2 | ENST00000359543.7 | No | TCGA-HQ-A5NE-01 | 2339537 | p.S105L | c.314C>T | Urinary tract | Bladder | NS | Carcinoma | NS | NS | - | 413 | Confirmed Somatic | Tumour Sample | Unknown | 16:10537930..10537930 |
| EMP2 | ENST00000359543.7 | No | TCGA-CD-A4MJ-01 | 2198030 | p.I100T | c.299T>C | Stomach | NS | NS | Carcinoma | Adenocarcinoma | NS | - | 541 | Confirmed Somatic | Unknown | Unknown | 16:10537945..10537945 |
| EMP2 | ENST00000359543.7 | No | 299LC | 2637238 | p.I100F | c.298A>T | Lung | NS | NS | Carcinoma | Squamous cell carcinoma | NS | 26503331 | - | Confirmed Somatic | Tumour Sample | Heterozygous | 16:10537946..10537946 |
| EMP2 | ENST00000359543.7 | No | TCGA-A3-A8CQ-01 | 2262754 | p.I100V | c.298A>G | Kidney | NS | NS | Carcinoma | Clear cell renal cell carcinoma | NS | - | 416 | Confirmed Somatic | Tumour Sample | Unknown | 16:10537946..10537946 |
| EMP2 | ENST00000359543.7 | No | TCGA-D3-A8GL-06 | 2263233 | p.S99F | c.296C>T | Skin | NS | NS | Malignant melanoma | NS | NS | - | 540 | Confirmed Somatic | Unknown | Unknown | 16:10537948..10537948 |
| EMP2 | ENST00000359543.7 | No | W33T | 2745056 | p.R94K | c.281G>A | Thyroid | NS | NS | Carcinoma | NS | NS | - | 676 | Confirmed Somatic | Tumour Sample | Unknown | 16:10537963..10537963 |
| EMP2 | ENST00000359543.7 | No | Thyroid-CN-WZ046T | 2635107 | p.R94K | c.281G>A | Thyroid | NS | NS | Carcinoma | NS | NS | - | 676 | Confirmed Somatic | Tumour Sample | Unknown | 16:10537963..10537963 |
| EMP2 | ENST00000359543.7 | No | PDA_085 | 2367426 | p.R88H | c.263G>A | Pancreas | NS | NS | Carcinoma | Ductal carcinoma | NS | 25855536 | - | Confirmed Somatic | Unknown | Unknown | 16:10537981..10537981 |
| EMP2 | ENST00000359543.7 | No | SNU-175 | 2302007 | p.R88H | c.263G>A | Large intestine | NS | NS | Carcinoma | Adenocarcinoma | NS | 24755471 | - | Previously Reported | Cultured | Heterozygous | 16:10537981..10537981 |
| EMP2 | ENST00000359543.7 | No | T3269 | 2658287 | p.R88C | c.262C>T | Large intestine | NS | NS | Carcinoma | Adenocarcinoma | NS | 27149842 | - | Confirmed Somatic | Unknown | Unknown | 16:10537982..10537982 |
| EMP2 | ENST00000359543.7 | No | HN_63048 | 1560589 | p.V83A | c.248T>C | Upper aerodigestive tract | Mouth | NS | Carcinoma | Squamous cell carcinoma | NS | 21798893 | - | Variant of unknown origin | Tumour Sample | Unknown | 16:10537996..10537996 |
| EMP2 | ENST00000359543.7 | No | ICGC_GBM85_tumor | 2634877 | p.F79Y | c.236T>A | Central nervous system | Brain | NS | Primitive neuroectodermal tumour-medulloblastoma | NS | NS | - | 379 | Confirmed Somatic | Tumour Sample | Unknown | 16:10538008..10538008 |
| EMP2 | ENST00000359543.7 | No | 587284 | 1766769 | p.A78T | c.232G>A | Large intestine | Colon | NS | Carcinoma | Adenocarcinoma | NS | 22895193 | - | Confirmed Somatic | Tumour Sample | Unknown | 16:10538012..10538012 |
| EMP2 | ENST00000359543.7 | No | TCGA-AX-A0J1-01 | 1783377 | p.A78T | c.232G>A | Endometrium | NS | NS | Carcinoma | Endometrioid carcinoma | NS | - | 419 | Previously Reported | Tumour Sample | Heterozygous | 16:10538012..10538012 |
| EMP2 | ENST00000359543.7 | No | TCGA-CC-A7IF-01 | 2340045 | p.C76F | c.227G>T | Liver | NS | NS | Carcinoma | Hepatocellular carcinoma | NS | - | 628 | Confirmed Somatic | Tumour Sample | Unknown | 16:10538017..10538017 |
| EMP2 | ENST00000359543.7 | No | PD9696a | 1655475 | p.A63S | c.187G>T | Breast | NS | NS | Carcinoma | Ductal carcinoma | NS | - | 652 | Confirmed Somatic | Tumour Sample | Unknown | 16:10538057..10538057 |
| EMP2 | ENST00000359543.7 | No | TCGA-HU-A4GT-01 | 2197914 | p.T60M | c.179C>T | Stomach | NS | NS | Carcinoma | Adenocarcinoma | NS | - | 541 | Confirmed Somatic | Unknown | Unknown | 16:10538065..10538065 |
| EMP2 | ENST00000359543.7 | No | TCGA-AA-3713-01 | 1651032 | p.S59Y | c.176C>A | Large intestine | Colon | Ascending | Carcinoma | Adenocarcinoma | NS | - | 376 | Confirmed Somatic | Unknown | Unknown | 16:10538068..10538068 |
| EMP2 | ENST00000359543.7 | No | TCGA-D1-A103-01 | 1783494 | p.S54I | c.161G>T | Endometrium | NS | NS | Carcinoma | Endometrioid carcinoma | NS | - | 419 | Variant of unknown origin | Tumour Sample | Heterozygous | 16:10543578..10543578 |
| EMP2 | ENST00000359543.7 | No | TCGA-B5-A1MR-01 | 2198376 | p.N45D | c.133A>G | Endometrium | NS | NS | Carcinoma | Endometrioid carcinoma | NS | - | 419 | Confirmed Somatic | Unknown | Unknown | 16:10543606..10543606 |
| EMP2 | ENST00000359543.7 | No | 2521252 | 2521252 | p.D37E | c.111T>G | Skin | Face | NS | Carcinoma | Basal cell carcinoma | NS | 25759019 | - | Confirmed Somatic | Tumour Sample | Unknown | 16:10543628..10543628 |
| EMP2 | ENST00000359543.7 | No | ESCC_BICR_040T | 2456895 | p.W29C | c.87G>C | Oesophagus | NS | NS | Carcinoma | Squamous cell carcinoma | NS | - | 582 | Confirmed Somatic | Tumour Sample | Unknown | 16:10543652..10543652 |
| EMP2 | ENST00000359543.7 | No | TCGA-D1-A163-01 | 1783501 | p.D25N | c.73G>A | Endometrium | NS | NS | Carcinoma | Endometrioid carcinoma | NS | - | 419 | Confirmed Somatic | Tumour Sample | Unknown | 16:10547545..10547545 |
| EMP2 | ENST00000359543.7 | No | HCC41 | 2120840 | p.V24F | c.70G>T | Liver | NS | NS | Carcinoma | NS | NS | - | 323 | Confirmed Somatic | Unknown | Unknown | 16:10547548..10547548 |
| EMP2 | ENST00000359543.7 | No | HCC41T | 1566756 | p.V24F | c.70G>T | Liver | NS | NS | Carcinoma | Hepatocellular carcinoma | NS | - | 323 | Previously Reported | Tumour Sample | Heterozygous | 16:10547548..10547548 |
| EMP2 | ENST00000359543.7 | No | HUB-02-B2-027 | 2607091 | p.I13N | c.38T>A | Large intestine | NS | NS | Carcinoma | Adenocarcinoma | NS | - | 670 | Confirmed Somatic | Cultured | Unknown | 16:10547580..10547580 |
| EMP2 | ENST00000359543.7 | No | CAKI-1 | 1998436 | p.A10T | c.28G>A | Kidney | NS | NS | Carcinoma | Clear cell renal cell carcinoma | NS | 23856246 | - | Confirmed Somatic | Cultured | Heterozygous | 16:10547590..10547590 |
| EMP2 | ENST00000359543.7 | No | TCGA-AP-A051-01 | 1783330 | p.A10T | c.28G>A | Endometrium | NS | NS | Carcinoma | Endometrioid carcinoma | NS | - | 419 | Previously Reported | Tumour Sample | Heterozygous | 16:10547590..10547590 |
| EMP2 | ENST00000359543.7 | No | TCGA-AD-6895-01 | 1651144 | p.A10T | c.28G>A | Large intestine | Caecum | NS | Carcinoma | Adenocarcinoma | NS | - | 376 | Confirmed Somatic | Unknown | Unknown | 16:10547590..10547590 |
| EMP2 | ENST00000359543.7 | No | T233366 | 2658251 | p.A10T | c.28G>A | Large intestine | NS | NS | Carcinoma | Adenocarcinoma | NS | 27149842 | - | Confirmed Somatic | Unknown | Unknown | 16:10547590..10547590 |
| EMP2 | ENST00000359543.7 | No | PD42092a | 2894833 | p.L5F | c.13C>T | Skin | Head neck | NS | Malignant melanoma | Superficial spreading | NS | 33024263 | - | Variant of unknown origin | Tumour Sample | Unknown | 16:10547605..10547605 |
| EMP3 | ENST00000270221.10 | No | TCGA-97-7938-01 | 1914111 | p.V7L | c.18_19delinsTT | Lung | Right upper lobe | NS | Carcinoma | Adenocarcinoma | NS | - | 417 | Variant of unknown origin | Tumour Sample | Unknown | 19:48326862..48326863 |
| EMP3 | ENST00000270221.10 | No | TCGA-97-7938-01 | 1914111 | p.V7L | c.19G>T | Lung | Right upper lobe | NS | Carcinoma | Adenocarcinoma | NS | - | 417 | Confirmed Somatic | Tumour Sample | Unknown | 19:48326863..48326863 |
| EMP3 | ENST00000270221.10 | No | BK0014 | 2237568 | p.S9L | c.26C>T | Kidney | NS | NS | Carcinoma | Clear cell renal cell carcinoma | NS | 24504440 | - | Confirmed Somatic | Tumour Sample | Unknown | 19:48326870..48326870 |
| EMP3 | ENST00000270221.10 | No | OSCC-GB_00660111 | 2340479 | p.A10S | c.28G>T | Upper aerodigestive tract | Mouth | NS | Carcinoma | NS | NS | - | 539 | Confirmed Somatic | Tumour Sample | Unknown | 19:48326872..48326872 |
| EMP3 | ENST00000270221.10 | No | CSCC-29-T | 2292470 | p.L16F | c.46C>T | Skin | Head neck | NS | Carcinoma | Squamous cell carcinoma | NS | 25303977 | - | Confirmed Somatic | Tumour Sample | Heterozygous | 19:48326890..48326890 |
| EMP3 | ENST00000270221.10 | No | TCGA-EA-A50E-01 | 2193351 | p.L16V | c.46C>G | Cervix | NS | NS | Carcinoma | Squamous cell carcinoma | NS | - | 415 | Confirmed Somatic | Unknown | Unknown | 19:48326890..48326890 |
| EMP3 | ENST00000270221.10 | No | T3690 | 2658266 | p.I17M | c.51A>G | Large intestine | NS | NS | Carcinoma | Adenocarcinoma | NS | 27149842 | - | Confirmed Somatic | Unknown | Unknown | 19:48326895..48326895 |
| EMP3 | ENST00000270221.10 | No | 587298 | 1766774 | p.V21M | c.61G>A | Large intestine | Colon | NS | Carcinoma | Adenocarcinoma | NS | 22895193 | - | Confirmed Somatic | Tumour Sample | Unknown | 19:48326905..48326905 |
| EMP3 | ENST00000270221.10 | No | 2014_SWES_20_DX | 2743831 | p.A22T | c.64G>A | Haematopoietic and lymphoid | NS | NS | Haematopoietic neoplasm | Acute myeloid leukaemia | NS | - | 544 | Confirmed Somatic | Tumour Sample | Unknown | 19:48326908..48326908 |
| EMP3 | ENST00000270221.10 | No | TCGA-66-2768-01 | 1782691 | p.A22T | c.64G>A | Lung | NS | NS | Carcinoma | Squamous cell carcinoma | NS | - | 418 | Confirmed Somatic | Tumour Sample | Unknown | 19:48326908..48326908 |
| EMP3 | ENST00000270221.10 | No | TCGA-86-8585-01 | 2194834 | p.P32L | c.95C>T | Lung | NS | NS | Carcinoma | Adenocarcinoma | NS | - | 417 | Confirmed Somatic | Unknown | Unknown | 19:48327537..48327537 |
| EMP3 | ENST00000270221.10 | No | TCGA-D3-A2J9-06 | 2121512 | p.P32H | c.95C>A | Skin | NS | NS | Malignant melanoma | NS | NS | - | 540 | Confirmed Somatic | Unknown | Unknown | 19:48327537..48327537 |
| EMP3 | ENST00000270221.10 | No | TCGA-D3-A2J8-06 | 2121511 | p.P32H | c.95C>A | Skin | NS | NS | Malignant melanoma | NS | NS | - | 540 | Confirmed Somatic | Unknown | Unknown | 19:48327537..48327537 |
| EMP3 | ENST00000270221.10 | No | CHG-13-29153T | 2634325 | p.G33W | c.97G>T | Liver | NS | NS | Other | Neoplasm | NS | - | 660 | Confirmed Somatic | Tumour Sample | Unknown | 19:48327539..48327539 |
| EMP3 | ENST00000270221.10 | No | TCGA-D3-A8GP-06 | 2340115 | p.E35K | c.103G>A | Skin | NS | NS | Malignant melanoma | NS | NS | - | 540 | Confirmed Somatic | Unknown | Unknown | 19:48327545..48327545 |
| EMP3 | ENST00000270221.10 | No | 578 | 2131838 | p.D42N | c.124G>A | Upper aerodigestive tract | Mouth | NS | Carcinoma | Squamous cell carcinoma | NS | 23619168 | - | Confirmed Somatic | Tumour Sample | Unknown | 19:48327566..48327566 |
| EMP3 | ENST00000270221.10 | No | TCGA-CV-6952-01 | 2193532 | p.D42N | c.124G>A | Upper aerodigestive tract | Head neck | NS | Carcinoma | Squamous cell carcinoma | NS | - | 627 | Confirmed Somatic | Unknown | Unknown | 19:48327566..48327566 |
| EMP3 | ENST00000270221.10 | No | TCGA-AX-A0J0-01 | 1783376 | p.C43R | c.127T>C | Endometrium | NS | NS | Carcinoma | Endometrioid carcinoma | NS | - | 419 | Variant of unknown origin | Tumour Sample | Heterozygous | 19:48327569..48327569 |
| EMP3 | ENST00000270221.10 | No | T2992 | 2658437 | p.D48N | c.142G>A | Large intestine | NS | NS | Carcinoma | Adenocarcinoma | NS | 27149842 | - | Confirmed Somatic | Unknown | Unknown | 19:48327584..48327584 |
| EMP3 | ENST00000270221.10 | No | SC_9008 | 2467356 | p.K64N | c.192G>T | Prostate | NS | NS | Carcinoma | NS | NS | 26000489 | - | Confirmed Somatic | Tumour Sample | Heterozygous | 19:48329362..48329362 |
| EMP3 | ENST00000270221.10 | No | NUGC-4 | 2807639 | p.A65V | c.194C>T | Stomach | NS | NS | Carcinoma | Adenocarcinoma | NS | 24807215 | - | Previously Reported | Cultured | Unknown | 19:48329364..48329364 |
| EMP3 | ENST00000270221.10 | No | SJHGG003_A | 2307333 | p.A65V | c.194C>T | Central nervous system | Brainstem | NS | Glioma | Astrocytoma Grade IV | Glioblastoma multiforme | 24705251 | - | Confirmed Somatic | Tumour Sample | Unknown | 19:48329364..48329364 |
| EMP3 | ENST00000270221.10 | No | ESO-717 | 1890958 | p.A65V | c.194C>T | Oesophagus | NS | NS | Carcinoma | Adenocarcinoma | NS | 23525077 | - | Previously Reported | Tumour Sample | Unknown | 19:48329364..48329364 |
| EMP3 | ENST00000270221.10 | No | TCGA-MP-A4TF-01 | 2194749 | p.Q67L | c.200A>T | Lung | NS | NS | Carcinoma | Adenocarcinoma | NS | - | 417 | Confirmed Somatic | Unknown | Unknown | 19:48329370..48329370 |
| EMP3 | ENST00000270221.10 | No | 5-VS021-T1 | 2869841 | p.L69F | c.205C>T | Skin | NS | NS | Carcinoma | Basal cell carcinoma | NS | 26950094 | - | Confirmed Somatic | Tumour Sample | Unknown | 19:48329375..48329375 |
| EMP3 | ENST00000270221.10 | No | RMS77 | 2355925 | p.M70L | c.208A>T | Soft tissue | Striated muscle | Face | Rhabdomyosarcoma | Embryonal | NS | 24793135 | - | Variant of unknown origin | Tumour Sample | Unknown | 19:48329378..48329378 |
| EMP3 | ENST00000270221.10 | No | TCGA-D5-6540-01 | 1651258 | p.M70V | c.208A>G | Large intestine | Caecum | NS | Carcinoma | Adenocarcinoma | NS | - | 376 | Confirmed Somatic | Unknown | Unknown | 19:48329378..48329378 |
| EMP3 | ENST00000270221.10 | No | T578 | 2296255 | p.R92Q | c.275G>A | Large intestine | Caecum | NS | Carcinoma | Adenocarcinoma | NS | 25344691 | - | Confirmed Somatic | Unknown | Unknown | 19:48329445..48329445 |
| EMP3 | ENST00000270221.10 | No | T578 | 2658233 | p.R92Q | c.275G>A | Large intestine | NS | NS | Carcinoma | Adenocarcinoma | NS | 27149842 | - | Confirmed Somatic | Unknown | Unknown | 19:48329445..48329445 |
| EMP3 | ENST00000270221.10 | No | CHG-2014-22138T | 2634361 | p.A99V | c.296C>T | Liver | NS | NS | Other | Neoplasm | NS | - | 660 | Confirmed Somatic | Tumour Sample | Unknown | 19:48329466..48329466 |
| EMP3 | ENST00000270221.10 | No | GCTK_394_T | 2747548 | p.L102P | c.305T>C | Stomach | NS | NS | Carcinoma | NS | NS | - | 683 | Confirmed Somatic | Tumour Sample | Unknown | 19:48329475..48329475 |
| EMP3 | ENST00000270221.10 | No | 19 | 2640423 | p.A119T | c.355G>A | Lung | NS | NS | Carcinoma | Adenocarcinoma | NS | 27545006 | - | Confirmed Somatic | Tumour Sample | Unknown | 19:48330333..48330333 |
| EMP3 | ENST00000270221.10 | No | TCGA-FB-AAQ0-01 | 2339678 | p.A119G | c.356C>G | Pancreas | NS | NS | Carcinoma | Ductal carcinoma | NS | - | 629 | Confirmed Somatic | Tumour Sample | Unknown | 19:48330334..48330334 |
| EMP3 | ENST00000270221.10 | No | 1T26 | 2745666 | p.H121N | c.361C>A | Oesophagus | NS | NS | Carcinoma | NS | NS | - | 582 | Confirmed Somatic | Tumour Sample | Unknown | 19:48330339..48330339 |
| EMP3 | ENST00000270221.10 | No | 1N26-VS-1T26 | 2582855 | p.H121N | c.361C>A | Oesophagus | NS | NS | Carcinoma | Squamous cell carcinoma | NS | 26759717 | - | Confirmed Somatic | Tumour Sample | Unknown | 19:48330339..48330339 |
| EMP3 | ENST00000270221.10 | No | 1N26-VS-1T26 | 2363538 | p.H121N | c.361C>A | Oesophagus | NS | NS | Carcinoma | Squamous cell carcinoma | NS | 25839328 | - | Confirmed Somatic | Unknown | Unknown | 19:48330339..48330339 |
| EMP3 | ENST00000270221.10 | No | A549 | 2776223 | p.I125V | c.373A>G | Lung | NS | NS | Carcinoma | Adenocarcinoma | NS | 29681454 | - | Variant of unknown origin | Unknown | Unknown | 19:48330351..48330351 |
| EMP3 | ENST00000270221.10 | No | H2286 | 2776348 | p.I125V | c.373A>G | Lung | NS | NS | Carcinoma | Non small cell carcinoma | NS | 29681454 | - | Variant of unknown origin | Unknown | Unknown | 19:48330351..48330351 |
| EMP3 | ENST00000270221.10 | No | H1568 | 2776237 | p.I125V | c.373A>G | Lung | NS | NS | Carcinoma | Adenocarcinoma | NS | 29681454 | - | Variant of unknown origin | Unknown | Unknown | 19:48330351..48330351 |
| EMP3 | ENST00000270221.10 | No | CALU1 | 2776225 | p.I125V | c.373A>G | Lung | NS | NS | Carcinoma | Squamous cell carcinoma | NS | 29681454 | - | Variant of unknown origin | Unknown | Unknown | 19:48330351..48330351 |
| EMP3 | ENST00000270221.10 | No | TCGA-AX-A2HD-01 | 2198411 | p.G132E | c.395G>A | Endometrium | NS | NS | Carcinoma | Endometrioid carcinoma | NS | - | 419 | Confirmed Somatic | Unknown | Unknown | 19:48330373..48330373 |
| EMP3 | ENST00000270221.10 | No | 13280 | 2478831 | p.G136E | c.407G>A | Lung | NS | NS | Carcinoma | Squamous cell carcinoma | NS | 22510280 | - | Confirmed Somatic | Tumour Sample | Unknown | 19:48330385..48330385 |
| EMP3 | ENST00000270221.10 | No | TCGA-37-A5EL-01 | 2457464 | p.Y137C | c.410A>G | Lung | NS | NS | Carcinoma | Squamous cell carcinoma | NS | - | 418 | Confirmed Somatic | Tumour Sample | Unknown | 19:48330388..48330388 |
| EMP3 | ENST00000270221.10 | No | LS180 | 2301998 | p.C138R | c.412T>C | Large intestine | NS | NS | Carcinoma | Adenocarcinoma | NS | 24755471 | - | Variant of unknown origin | Cultured | Heterozygous | 19:48330390..48330390 |
| EMP3 | ENST00000270221.10 | No | LS174T | 2301997 | p.C138R | c.412T>C | Large intestine | NS | NS | Carcinoma | Adenocarcinoma | NS | 24755471 | - | Variant of unknown origin | Cultured | Heterozygous | 19:48330390..48330390 |
| EMP3 | ENST00000270221.10 | No | 44229 | 2746097 | p.F139L | c.415T>C | Large intestine | NS | NS | Carcinoma | Adenocarcinoma | NS | - | 646 | Confirmed Somatic | Tumour Sample | Unknown | 19:48330393..48330393 |
| EMP3 | ENST00000270221.10 | No | 19 | 2693102 | p.W143L | c.428G>T | Central nervous system | Brain | NS | Glioma | Oligodendroglioma Grade III | Anaplastic | 28270234 | - | Confirmed Somatic | Tumour Sample | Unknown | 19:48330406..48330406 |
| EMP3 | ENST00000270221.10 | No | TCGA-D3-A3C3-06 | 2121527 | p.G153S | c.457G>A | Skin | NS | NS | Malignant melanoma | NS | NS | - | 540 | Confirmed Somatic | Unknown | Unknown | 19:48330435..48330435 |
| EMP3 | ENST00000270221.10 | No | TCGA-BF-A1Q0-01 | 2121497 | p.H158N | c.472C>A | Skin | NS | NS | Malignant melanoma | NS | NS | - | 540 | Confirmed Somatic | Unknown | Unknown | 19:48330450..48330450 |
| EMP3 | ENST00000270221.10 | No | TCGA-CM-5861-01 | 1651218 | p.L159P | c.476T>C | Large intestine | Caecum | NS | Carcinoma | Adenocarcinoma | NS | - | 376 | Confirmed Somatic | Unknown | Unknown | 19:48330454..48330454 |
| PMP22 | ENST00000312280.8 | No | 230 | 2146914 | p.R157W | c.469C>T | Stomach | Gastroesophageal junction | NS | Carcinoma | Adenocarcinoma | NS | 24308032 | - | Confirmed Somatic | Tumour Sample | Unknown | 17:15230931..15230931 |
| PMP22 | ENST00000312280.8 | No | TCGA-DK-A2I4-01 | 2193288 | p.S46F | c.137C>T | Urinary tract | Bladder | NS | Carcinoma | NS | NS | - | 413 | Confirmed Somatic | Unknown | Unknown | 17:15259135..15259135 |
| PMP22 | ENST00000312280.8 | No | TCGA-XF-AAMX-01 | 2339188 | p.S47L | c.140C>T | Urinary tract | Bladder | NS | Carcinoma | NS | NS | - | 413 | Confirmed Somatic | Tumour Sample | Unknown | 17:15259132..15259132 |
| PMP22 | ENST00000312280.8 | No | TCGA-BH-A18G-01 | 2187796 | p.A35T | c.103G>A | Breast | NS | NS | Carcinoma | NS | NS | - | 414 | Confirmed Somatic | Unknown | Unknown | 17:15259169..15259169 |
| PMP22 | ENST00000312280.8 | No | T3202 | 2296197 | p.A35T | c.103G>A | Large intestine | Caecum | NS | Carcinoma | Adenocarcinoma | NS | 25344691 | - | Confirmed Somatic | Unknown | Unknown | 17:15259169..15259169 |
| PMP22 | ENST00000312280.8 | No | TCGA-G4-6302-01 | 1651321 | p.A35T | c.103G>A | Large intestine | Caecum | NS | Carcinoma | Adenocarcinoma | NS | - | 376 | Confirmed Somatic | Unknown | Unknown | 17:15259169..15259169 |
| PMP22 | ENST00000312280.8 | No | TCGA-AZ-6598-01 | 1651172 | p.G48E | c.143G>A | Large intestine | Caecum | NS | Carcinoma | Adenocarcinoma | NS | - | 376 | Confirmed Somatic | Unknown | Unknown | 17:15259129..15259129 |
| PMP22 | ENST00000312280.8 | No | TCGA-IR-A3LH-01 | 2193368 | p.F83L | c.249C>G | Cervix | NS | NS | Carcinoma | Squamous cell carcinoma | NS | - | 415 | Confirmed Somatic | Unknown | Unknown | 17:15239541..15239541 |
| PMP22 | ENST00000312280.8 | No | TCGA-AA-3811-01 | 1651034 | p.H121Y | c.361C>T | Large intestine | Colon | Ascending | Carcinoma | Adenocarcinoma | NS | - | 376 | Confirmed Somatic | Unknown | Unknown | 17:15231039..15231039 |
| PMP22 | ENST00000312280.8 | No | 587332 | 1766784 | p.V119A | c.356T>C | Large intestine | Colon | NS | Carcinoma | Adenocarcinoma | NS | 22895193 | - | Confirmed Somatic | Tumour Sample | Unknown | 17:15231044..15231044 |
| PMP22 | ENST00000312280.8 | No | T3202 | 2658288 | p.A35T | c.103G>A | Large intestine | NS | NS | Carcinoma | Adenocarcinoma | NS | 27149842 | - | Confirmed Somatic | Unknown | Unknown | 17:15259169..15259169 |
| PMP22 | ENST00000312280.8 | No | T4430 | 2658280 | p.A35T | c.103G>A | Large intestine | NS | NS | Carcinoma | Adenocarcinoma | NS | 27149842 | - | Confirmed Somatic | Unknown | Unknown | 17:15259169..15259169 |
| PMP22 | ENST00000312280.8 | No | sysucc-311T | 2456736 | p.V50I | c.148G>A | Large intestine | NS | NS | Carcinoma | Adenocarcinoma | NS | - | 646 | Confirmed Somatic | Tumour Sample | Unknown | 17:15259124..15259124 |
| PMP22 | ENST00000312280.8 | No | 7C | 2549418 | p.A135T | c.403G>A | Large intestine | NS | NS | Carcinoma | Adenocarcinoma | NS | - | 646 | Confirmed Somatic | Tumour Sample | Unknown | 17:15230997..15230997 |
| PMP22 | ENST00000312280.8 | No | 8067938 | 2263294 | p.W28C | c.84G>T | Pancreas | NS | NS | Carcinoma | Ductal carcinoma | NS | - | 328 | Confirmed Somatic | Tumour Sample | Unknown | 17:15259188..15259188 |
| PMP22 | ENST00000312280.8 | No | PCSI_0084_Pa_P_526 | 2197232 | p.S45F | c.134C>T | Pancreas | NS | NS | Carcinoma | Ductal carcinoma | NS | - | 382 | Confirmed Somatic | Unknown | Unknown | 17:15259138..15259138 |
| PMP22 | ENST00000312280.8 | No | PCSI_0084_Pa_X | 2121294 | p.S45F | c.134C>T | Pancreas | NS | NS | Carcinoma | Ductal carcinoma | NS | - | 382 | Confirmed Somatic | Unknown | Unknown | 17:15259138..15259138 |
| PMP22 | ENST00000312280.8 | No | TCGA-D1-A176-01 | 1783521 | p.S64Y | c.191C>A | Endometrium | NS | NS | Carcinoma | Endometrioid carcinoma | NS | - | 419 | Confirmed Somatic | Tumour Sample | Unknown | 17:15239599..15239599 |
| PMP22 | ENST00000312280.8 | No | TCGA-CQ-7071-01 | 2193562 | p.R159C | c.475C>T | Upper aerodigestive tract | Head neck | NS | Carcinoma | Squamous cell carcinoma | NS | - | 627 | Confirmed Somatic | Unknown | Unknown | 17:15230925..15230925 |
| PMP22 | ENST00000312280.8 | No | TCGA-CJ-4920-01 | 1779983 | p.T68A | c.202A>G | Kidney | NS | NS | Carcinoma | Clear cell renal cell carcinoma | NS | - | 416 | Confirmed Somatic | Tumour Sample | Unknown | 17:15239588..15239588 |
| PMP22 | ENST00000312280.8 | No | TCGA-B4-5836-01 | 1913402 | p.R157W | c.469C>T | Kidney | NS | NS | Carcinoma | Clear cell renal cell carcinoma | NS | - | 416 | Previously Reported | Tumour Sample | Unknown | 17:15230931..15230931 |
| PMP22 | ENST00000312280.8 | No | RK308_C01 | 2194686 | p.A35T | c.103G>A | Liver | NS | NS | Carcinoma | NS | NS | - | 322 | Confirmed Somatic | Unknown | Unknown | 17:15259169..15259169 |
| PMP22 | ENST00000312280.8 | No | CHG-12-27106T | 2634303 | p.I8M | c.24C>G | Liver | NS | NS | Other | Neoplasm | NS | - | 660 | Confirmed Somatic | Tumour Sample | Unknown | 17:15260704..15260704 |
| PMP22 | ENST00000312280.8 | No | TCGA-RC-A6M4-01 | 2457488 | p.S45P | c.133T>C | Liver | NS | NS | Carcinoma | Hepatocellular carcinoma | NS | - | 628 | Confirmed Somatic | Tumour Sample | Unknown | 17:15259139..15259139 |
| PMP22 | ENST00000312280.8 | No | TCGA-CC-A9FW-01 | 2386071 | p.R157Q | c.470G>A | Liver | NS | NS | Carcinoma | Hepatocellular carcinoma | NS | - | 628 | Confirmed Somatic | Tumour Sample | Unknown | 17:15230930..15230930 |
| PMP22 | ENST00000312280.8 | No | H2009 | 2776255 | p.S57L | c.170C>T | Lung | NS | NS | Carcinoma | Adenocarcinoma | NS | 29681454 | - | Confirmed Somatic | Unknown | Unknown | 17:15259102..15259102 |
| PMP22 | ENST00000312280.8 | No | LUAD-NYU330 | 1765168 | p.G94S | c.280G>A | Lung | NS | NS | Carcinoma | Adenocarcinoma | NS | 22980975 | - | Variant of unknown origin | Tumour Sample | Unknown | 17:15239510..15239510 |
| PMP22 | ENST00000312280.8 | No | IGC-04-1055 | 2662294 | p.C109W | c.327C>G | Lung | NS | NS | Carcinoma | Adenocarcinoma | NS | 27923066 | - | Confirmed Somatic | Tumour Sample | Unknown | 17:15231073..15231073 |
| PMP22 | ENST00000312280.8 | No | LUAD-YINHD | 1765263 | p.A113S | c.337G>T | Lung | NS | NS | Carcinoma | Adenocarcinoma | NS | 22980975 | - | Previously Reported | Tumour Sample | Unknown | 17:15231063..15231063 |
| PMP22 | ENST00000312280.8 | No | TCGA-96-7545-01 | 2195198 | p.S45P | c.133T>C | Lung | NS | NS | Carcinoma | Squamous cell carcinoma | NS | - | 418 | Confirmed Somatic | Unknown | Unknown | 17:15259139..15259139 |
| PMP22 | ENST00000312280.8 | No | S00837 | 1759200 | p.K92R | c.275A>G | Lung | NS | NS | Carcinoma | Small cell carcinoma | NS | 22941188 | - | Variant of unknown origin | Tumour Sample | Unknown | 17:15239515..15239515 |
| PMP22 | ENST00000312280.8 | No | 585260 | 1759403 | p.H121Q | c.363C>G | Lung | NS | NS | Carcinoma | Small cell carcinoma | NS | 22941189 | - | Confirmed Somatic | Tumour Sample | Unknown | 17:15231037..15231037 |
| PMP22 | ENST00000312280.8 | No | 11 | 2640415 | p.K92E | c.274A>G | Lung | NS | NS | Carcinoma | Bronchioloalveolar adenocarcinoma | NS | 27545006 | - | Confirmed Somatic | Unknown | Unknown | 17:15239516..15239516 |
| PMP22 | ENST00000312280.8 | No | TCGA-55-6969-01 | 1914000 | p.W124C | c.372G>T | Lung | Right upper lobe | NS | Carcinoma | Adenocarcinoma | NS | - | 417 | Variant of unknown origin | Tumour Sample | Unknown | 17:15231028..15231028 |
| PMP22 | ENST00000312280.8 | No | AOCS-150-3-1 | 2186514 | p.L18V | c.52C>G | Ovary | NS | NS | Carcinoma | Mixed adenosquamous carcinoma | NS | - | 585 | Confirmed Somatic | Unknown | Unknown | 17:15260676..15260676 |
| PMP22 | ENST00000312280.8 | No | AOCS-150-8-X | 2186430 | p.L18V | c.52C>G | Ovary | NS | NS | Carcinoma | Mixed adenosquamous carcinoma | NS | - | 585 | Confirmed Somatic | Unknown | Unknown | 17:15260676..15260676 |
| PMP22 | ENST00000312280.8 | No | Pat_45_B | 2492884 | p.P144L | c.431C>T | NS | NS | NS | Malignant melanoma | NS | NS | 24265153 | - | Confirmed Somatic | Tumour Sample | Unknown | 17:15230969..15230969 |
| PMP22 | ENST00000312280.8 | No | Pat_53_B | 2492893 | p.R159C | c.475C>T | NS | NS | NS | Malignant melanoma | NS | NS | 24265153 | - | Confirmed Somatic | Tumour Sample | Unknown | 17:15230925..15230925 |
| PMP22 | ENST00000312280.8 | No | CPCG0092-F1 | 2688244 | p.A114V | c.341C>T | Prostate | NS | NS | Carcinoma | Adenocarcinoma | NS | - | 537 | Confirmed Somatic | Tumour Sample | Unknown | 17:15231059..15231059 |
| PMP22 | ENST00000312280.8 | No | 5-VS033-T1 | 2869854 | p.P144L | c.431C>T | Skin | NS | NS | Carcinoma | Basal cell carcinoma | NS | 26950094 | - | Confirmed Somatic | Tumour Sample | Unknown | 17:15230969..15230969 |
| PMP22 | ENST00000312280.8 | No | TCGA-D3-A2JC-06 | 2121515 | p.W61L | c.182G>T | Skin | NS | NS | Malignant melanoma | NS | NS | - | 540 | Confirmed Somatic | Unknown | Unknown | 17:15239608..15239608 |
| PMP22 | ENST00000312280.8 | No | PD42094c | 2894837 | p.V15M | c.43G>A | Skin | Trunk | NS | Malignant melanoma | Superficial spreading | NS | 33024263 | - | Variant of unknown origin | Tumour Sample | Unknown | 17:15260685..15260685 |
| PMP22 | ENST00000312280.8 | No | TCGA-F1-6177-01 | 2198156 | p.S55P | c.163T>C | Stomach | NS | NS | Carcinoma | Adenocarcinoma | NS | - | 541 | Confirmed Somatic | Unknown | Unknown | 17:15259109..15259109 |
| PMP22 | ENST00000312280.8 | No | TCGA-HU-A4GQ-01 | 2198093 | p.V110A | c.329T>C | Stomach | NS | NS | Carcinoma | Adenocarcinoma | NS | - | 541 | Confirmed Somatic | Unknown | Unknown | 17:15231071..15231071 |
| PMP22 | ENST00000312280.8 | No | TCGA-CD-8535-01 | 2198025 | p.A113S | c.337G>T | Stomach | NS | NS | Carcinoma | Adenocarcinoma | NS | - | 541 | Confirmed Somatic | Unknown | Unknown | 17:15231063..15231063 |
| PMP22 | ENST00000312280.8 | No | 100031 | 2807608 | p.P144H | c.431C>A | Stomach | NS | NS | Carcinoma | Adenocarcinoma | NS | 24807215 | - | Confirmed Somatic | Tumour Sample | Unknown | 17:15230969..15230969 |
| PMP22 | ENST00000312280.8 | No | 2785973 | 2785973 | p.S128L | c.383C>T | Stomach | NS | NS | Adenoma | NS | NS | 27175599 | - | Confirmed Somatic | Tumour Sample | Unknown | 17:15231017..15231017 |
| PMP22 | ENST00000312280.8 | No | GCTK_424_T | 2747457 | p.N32I | c.95A>T | Stomach | NS | NS | Carcinoma | NS | NS | - | 683 | Confirmed Somatic | Tumour Sample | Unknown | 17:15259177..15259177 |
| PMP22 | ENST00000312280.8 | No | GCTK_304_T | 2747404 | p.G33V | c.98G>T | Stomach | NS | NS | Carcinoma | NS | NS | - | 683 | Confirmed Somatic | Tumour Sample | Unknown | 17:15259174..15259174 |
| PMP22 | ENST00000312280.8 | No | 276 | 2748248 | p.F96L | c.286T>C | Stomach | NS | NS | Carcinoma | NS | NS | - | 683 | Confirmed Somatic | Tumour Sample | Unknown | 17:15239504..15239504 |
| PMP22 | ENST00000312280.8 | No | 175 | 2748147 | p.S128L | c.383C>T | Stomach | NS | NS | Carcinoma | NS | NS | - | 683 | Confirmed Somatic | Tumour Sample | Unknown | 17:15231017..15231017 |
| PMP22 | ENST00000312280.8 | No | PD42114a | 2894823 | p.H121Y | c.361C>T | Skin | Head neck | NS | Malignant melanoma | Superficial spreading | NS | 33024263 | - | Confirmed Somatic | Tumour Sample | Unknown | 17:15231039..15231039 |
| PMP22 | ENST00000312280.8 | No | XG11 | 2809775 | p.S149N | c.446G>A | Haematopoietic and lymphoid | NS | NS | Lymphoid neoplasm | Plasma cell myeloma | NS | 30545397 | - | Variant of unknown origin | Cultured | Heterozygous | 17:15230954..15230954 |
| PMP22 | ENST00000312280.8 | No | I2L-P7-Tumor-Organoid | 2433490 | p.F134S | c.401T>C | Large intestine | Colon | Ascending | Carcinoma | Adenocarcinoma | NS | 25957691 | - | Confirmed Somatic | Unknown | Heterozygous | 17:15230999..15230999 |
| PMP22 | ENST00000312280.8 | No | I2L-P8-Tumor-Biopsy | 2433473 | p.A146V | c.437C>T | Large intestine | Colon | Descending | Carcinoma | Adenocarcinoma | NS | 25957691 | - | Confirmed Somatic | Unknown | Heterozygous | 17:15230963..15230963 |
| PMP22 | ENST00000312280.8 | No | I2L-P8-Tumor-Organoid | 2433491 | p.A146V | c.437C>T | Large intestine | Colon | Descending | Carcinoma | Adenocarcinoma | NS | 25957691 | - | Confirmed Somatic | Unknown | Heterozygous | 17:15230963..15230963 |
| PMP22 | ENST00000312280.8 | No | Gp2D | 2301973 | p.F83L | c.247T>C | Large intestine | NS | NS | Carcinoma | Adenocarcinoma | NS | 24755471 | - | Variant of unknown origin | Cultured | Heterozygous | 17:15239543..15239543 |
| PMP22 | ENST00000312280.8 | No | Gp2D | 2301973 | p.P122L | c.365C>T | Large intestine | NS | NS | Carcinoma | Adenocarcinoma | NS | 24755471 | - | Variant of unknown origin | Cultured | Heterozygous | 17:15231035..15231035 |
| PMP22 | ENST00000312280.8 | No | Gp5D | 2301974 | p.P122L | c.365C>T | Large intestine | NS | NS | Carcinoma | Adenocarcinoma | NS | 24755471 | - | Variant of unknown origin | Cultured | Heterozygous | 17:15231035..15231035 |
| PMP22 | ENST00000312280.8 | No | HT115 | 2301987 | p.R159C | c.475C>T | Large intestine | NS | NS | Carcinoma | Adenocarcinoma | NS | 24755471 | - | Previously Reported | Cultured | Heterozygous | 17:15230925..15230925 |
| PMP22 | ENST00000312280.8 | No | TCGA-AP-A051-01 | 1783330 | p.R159C | c.475C>T | Endometrium | NS | NS | Carcinoma | Endometrioid carcinoma | NS | - | 419 | Previously Reported | Tumour Sample | Heterozygous | 17:15230925..15230925 |
| PMP22 | ENST00000312280.8 | No | MSK-PCa4_organoid | 2465138 | p.I101L | c.301A>C | Prostate | NS | NS | Carcinoma | Adenocarcinoma | NS | 25201530 | - | Confirmed Somatic | Cultured | Heterozygous | 17:15239489..15239489 |
| PMP22 | ENST00000312280.8 | No | SC_9096 | 2467418 | p.F143S | c.428T>C | Prostate | NS | NS | Carcinoma | NS | NS | 26000489 | - | Confirmed Somatic | Tumour Sample | Heterozygous | 17:15230972..15230972 |
| PMP22 | ENST00000312280.8 | No | SC_9099 | 2467420 | p.F143S | c.428T>C | Prostate | NS | NS | Carcinoma | NS | NS | 26000489 | - | Confirmed Somatic | Tumour Sample | Heterozygous | 17:15230972..15230972 |
